# Nerylneryl diphosphate is the precursor of serrulatane, viscidane and cembrane-type diterpenoids in *Eremophila* species

**DOI:** 10.1186/s12870-020-2293-x

**Published:** 2020-02-28

**Authors:** Oliver Gericke, Nikolaj Lervad Hansen, Gustav Blichfeldt Pedersen, Louise Kjaerulff, Dan Luo, Dan Staerk, Birger Lindberg Møller, Irini Pateraki, Allison Maree Heskes

**Affiliations:** 1 Plant Biochemistry Laboratory, Department of Plant and Environmental Sciences, University of Copenhagen, Thorvaldsensvej 40, DK-1871 Frederiksberg C, Denmark; 2 Center for Synthetic Biology “bioSYNergy”, Department of Plant and Environmental Sciences, University of Copenhagen, Thorvaldsensvej 40, DK-1871 Frederiksberg C, Denmark; 3 Department of Drug Design and Pharmacology, Faculty of Health and Medical Sciences, University of Copenhagen, DK-2100 Copenhagen, Denmark

**Keywords:** Bioactive diterpenoids, *Eremophila*, *cis*-prenyltransferase, Terpene synthase, Serrulatanes, Viscidanes, Plant natural products

## Abstract

**Background:**

*Eremophila* R.Br. (Scrophulariaceae) is a diverse genus of plants with species distributed across semi-arid and arid Australia. It is an ecologically important genus that also holds cultural significance for many Indigenous Australians who traditionally use several species as sources of medicines. Structurally unusual diterpenoids, particularly serrulatane and viscidane-types, feature prominently in the chemical profile of many species and recent studies indicate that these compounds are responsible for much of the reported bioactivity. We have investigated the biosynthesis of diterpenoids in three species: *Eremophila lucida*, *Eremophila drummondii* and *Eremophila denticulata* subsp. *trisulcata*.

**Results:**

In all studied species diterpenoids were localised to the leaf surface and associated with the occurrence of glandular trichomes. Trichome-enriched transcriptome databases were generated and mined for candidate terpene synthases (TPS). Four TPSs with diterpene biosynthesis activity were identified: *El*TPS31 and *El*TPS3 from *E. lucida* were found to produce (3*Z*,7*Z*,11*Z*)-cembratrien-15-ol and 5-hydroxyviscidane, respectively, and *Ed*TPS22 and *Edt*TPS4, from *E. drummondii* and *E. denticulata* subsp. *trisulcata,* respectively, were found to produce 8,9-dihydroserrulat-14-ene which readily aromatized to serrulat-14-ene. In all cases, the identified TPSs used the *cisoid* substrate, nerylneryl diphosphate (NNPP), to form the observed products. Subsequently, *cis*-prenyl transferases (CPTs) capable of making NNPP were identified in each species.

**Conclusions:**

We have elucidated two biosynthetic steps towards three of the major diterpene backbones found in this genus. Serrulatane and viscidane-type diterpenoids are promising candidates for new drug leads. The identification of an enzymatic route to their synthesis opens up the possibility of biotechnological production, making accessible a ready source of scaffolds for further modification and bioactivity testing.

## Background

*Eremophila* R.Br. (Scrophulariaceae) is a large and diverse genus of plants endemic to mainland Australia. Members of this genus occur across the continent with the greatest species diversity found in Western Australia [1]. Species range in form from prostrate ground covers to large shrubs and are found mainly in semi-arid to arid regions. *Eremophila* is an important source of traditional herbal medicines for many Indigenous Australians [2–5]. Although the species and methods for remedy preparation can differ between communities, leaves are the most frequently used plant part. They are used fresh or dried, boiled, pounded into pastes or mixed with oils to make therapeutic preparations used for treating a wide range of illnesses. Reported uses include treatments for skin and eye infections [2–4], fevers [3], pain [2–4], coughs and colds [2, 3, 5] gastrointestinal complaints [2, 3], and inflammation [3]. Investigations of the specific activity of selected *Eremophila* spp. extracts have found a range of different bioactivities including anti-viral [6], antibacterial [7–9], anti-cancer [10], and inhibition of ion channels [11]. Diterpenoids, particularly serrulatanes, have been identified as major sources of the observed bioactivity of many of the extracts and have been shown to possess antimalarial [12], antibacterial [13–17], anti-diabetic [18, 19] and anti-inflammatory [13] activities. Further reports on the bioactivity of structurally related diterpenoids isolated from *Leucophyllum frutescens* (also Scrophulariaceae) [20] and several marine gorgonian coral species [21] support this group of molecules as a potential source of new drug leads.

The diterpenoid chemistry of *Eremophila* is diverse with over 100 different structures reported to-date [12, 15, 17–19, 22]. Linear, macrocyclic, and polycyclic structures are represented across the genus, but no labdane-related diterpenoids (which are  often the predominant class found in plants [23]) have been reported. Instead, many of the diterpenoids appear to be C20 analogues of sesquiterpenes with an un-cyclized fourth prenyl unit. Because of their unusual structures and potential as drug leads, we set out to identify the enzymes involved in *Eremophila* diterpenoid biosynthesis.

Terpenes are biosynthesised from linear prenyl diphosphates of different lengths by enzymes belonging to the terpene synthase (TPS) family [24], which are classified into subfamilies based on phylogenetic relationships (TPS-a to TPS-h) [24–26]. The main pathway to diterpenoids in angiosperms involves the sequential activity of two TPSs (class II followed by class I) and leads to the formation of the labdane-related diterpenoids (characterised by a decalin core) [23]. Diterpenes can also be biosynthesised directly from geranylgeranyl diphosphate (GGPP) or nerylneryl diphosphate (NNPP, the all *cis* isomer of GGPP) by class I TPSs alone to generate linear [27, 28], macrocyclic [29–31] and (poly) cyclic [32–35] products. These enzymes catalyse metal ion dependant ionization of the diphosphate bond of their prenyl diphosphate substrates to generate a reactive carbocation molecule. This intermediate then undergoes a series of rearrangements (e.g. hydride and alkyl shifts, proton transfers, deprotonation and reprotonation) and/or cyclizations [36]. The specific pathways followed in these reaction cascades are dependent on the interactions of the substrate with active site residues and sometimes water molecules. Terpene products are then formed by either proton abstraction from the carbocation to yield diterpene olefins or by water quenching of the carbocation to yield hydroxylated diterpenoids.

The linear prenyl diphosphate precursors used by TPSs are derived from two isomeric C5 building blocks, isopentenyl diphosphate (IPP) and dimethylallyl diphosphate (DMAPP). In head to tail condensation reactions IPP is sequentially joined to DMAPP or an existing longer chain prenyl diphosphate acceptor to build prenyl diphosphates of different chain lengths. *Trans-*Prenyl diphosphates are the typical substrates of plant TPSs and are biosynthesised by different short-chain *trans*-prenyl transferases (*trans*-PTs) which generate products of specific chain lengths: geranyl diphosphate synthase (GPPS, C10), (*E*,*E*)-farnesyl diphosphate synthase ((*E*,*E*)-FPPS, C15), geranylgeranyl diphosphate synthase (GGPPS, C20) and the recently identified geranylfarnesyl diphosphate synthase (GFPPS, C25) [37, 38]. A limited number of plant TPSs are known to use *cis-*prenyl diphosphates as substrates [32, 34, 39–44]. *cis*-Prenyl diphosphates are biosynthesised by a family of enzymes known as *cis*-prenyl transferases (CPTs) which are evolutionarily unrelated to *trans*-PTs. They also biosynthesize prenyl diphosphates of different chain lengths, which are roughly defined as short (C10-C20), medium (C35-C55), long chain (C80-C95) and very-long-chain (> C50000) [45], the latter being involved in natural rubber biosynthesis [46–48]. Most reported CPTs produce medium and long-chain products like dolichols that are involved in core metabolic processes [45, 49]. A few studies report CPTs with short-chain biosynthesising activity involved in terpenoid biosynthetic pathways. For example, in *Solanum* spp. a group of short-chain CPTs has been identified that are involved in mono-, sesqui- and diterpene biosynthesis [32, 39, 40, 44, 50].

**Fig. 1:**
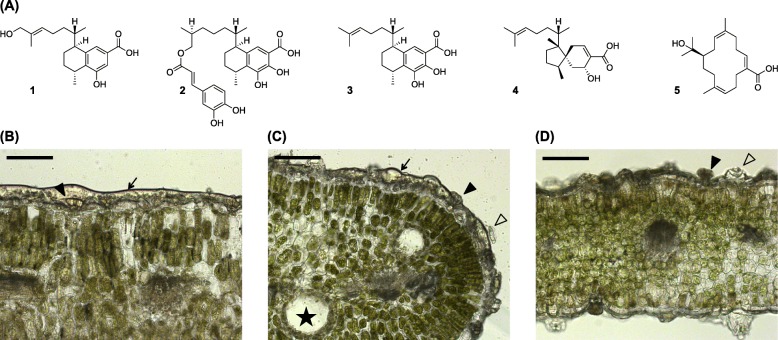
**a** Examples of diterpenoids reported from *E. denticulata* subsp. *trisulcata*: 8,17-dihydroxyserrulat-14-en-19-oic acid (**1**) [52]; *E. drummondii*: 7,8-dihydroxy-16-caffeoyloxyserrulat-19-oic acid (**2**), 7,8-dihydroxyserrulat-14-en-19-oic acid (**3**) [19]; *E. lucida*: 5-hydroxyviscida-3,14-dien-20-oic acid (**4**), (3*Z*, 7*E*, 11*Z*)-15-hydroxycembra-3,7,11-trien-19-oic acid (**5**) [18]. Bright field images of *Eremophila* spp. leaf cross sections: **b** *E. denticulata* subsp*. trisulcata*, (**c**) *E. drummondii* and (**d**) *E. lucida*. Arrows indicate resin layer coating the leaf surface, filled arrowheads indicate glandular trichomes, empty arrowheads indicate raised stomata and stars indicate internal oil glands. Scale bar = 100 μm

Apart from a single report investigating terpene biosynthesis in *Eremophila serrulata* in which two monoterpene synthases were identified and characterised as multifunctional myrcene/*Z*-(β)-ocimene synthases [51], no studies on the biosynthesis of terpenes in *Eremophila* are found in the literature. To investigate diterpenoid biosynthesis in *Eremophila* we selected three species with differing diterpenoid profiles for analysis: *E. lucida* Chinnock, *E. drummondii* F. Muell and *E. denticulata* subsp. *trisulcata* Chinnock. All species are endemic to southern Western Australia and grow to medium-sized shrubs on a range of soil types in open *Eucalyptus* woodlands [1]. The ethyl acetate leaf extract of *E. lucida* is dominated by two diterpenoids: a bicyclic viscidane, 5-hydroxyviscida-3,14-dien-20-oic acid (**4**; Fig. 1) and a macrocyclic compound, 15-hydroxycembra-3,7,11-trien-19-oic acid (**5**; Fig. 1) [18]. Coumpound **4** was identified as an inhibitor of protein-tyrosine phosphatase 1B (PTP1B), a potential target for type II diabetes therapeutics [18]. In contrast, the diterpenoid profiles of *E. drummondii* and *E. denticulata* subsp. *trisulcata* are characterised by the presence of serrulatanes (Fig. 1) [19, 53, 54], several of which from *E. drummondii* have also been reported to inhibit PTB1B as well as α-glucosidase [19].

Here, we describe the identification and functional characterisation of four TPSs that together account for the production of the three major diterpene backbones found across *E. lucida*, *E. drummondii* and *E. denticulata* subsp. *trisulcata.* In contrast to the majority of known plant diterpene synthases (diTPSs), they use the C20 *cisoid* precursor, nerylneryl diphosphate (NNPP), as substrate. Accordingly, we also identified one CPT in each species capable of producing NNPP.

## Results

The leaves of *E. lucida*, *E. denticulata subsp. trisulcata* and *E. drummundii* are covered by a resinous exudate (Fig. 1). Liquid chromatography-high resolution mass spectrometry

(LC-HRMS) analysis of this resinous layer showed the presence of *m*/*z* values expected of diterpenoids reported from these species (Fig. 1 and Additional file 2: Figure S1) [18, 19, 54]. Microscopic examination of leaves revealed the presence of peltate glandular trichomes on both the adaxial and abaxial leaf surfaces of all species. The trichomes were found to consist of a short stalk and a head of eight secretory cells, with combined diameter of 30–40 μm. Based on our results indicating surface-localisation of diterpenoids and in combination with the well-established role of glandular trichomes in terpenoid biosynthesis in numerous species [55], we speculated that they would also have this function in *Eremophila*. Consequently, the trichomes were targeted for transcriptomics.

Brushing and ice abrasion methods were not successful at removing the glandular trichomes from the leaf surface, presumably because of their embedded positioning in the epidermis and the large amounts of resinous exudate covering the leaf surface (Fig. 1). Consequently, a novel gland isolation procedure was developed. This procedure involved flash freezing of leaf material sandwiched between two plastic plates, followed by abrupt separation of the two plates. This resulted in resin and glandular trichomes sticking to the surface with minimal adherence of other leaf material. The material adhering to the plates was washed off with pre-chilled RNA isolation/lysis-buffer, the mixture collected, mechanically disrupted and finally extracted for RNA. The resulting RNA was used to generate trichome-enriched transcriptomes using Illumina HiSeq 2500 technology (for transcriptome statistics see Additional file 1: Table S1).

To get an initial indication of the activity of terpenoid biosynthesis in the trichomes, we searched for upstream genes involved in terpenoid metabolism from the mevalonate (MVA) and the 2-*C*-methyl-D-erythritol 4-phosphate (MEP) pathways using annotated *Arabidopsis* protein sequences as queries. In the trichome transcriptomes from all three *Eremophila* species transcripts representing genes from every step in the MVA and MEP pathways were detected (Additional file 1: Table S2).

**Fig. 2:**
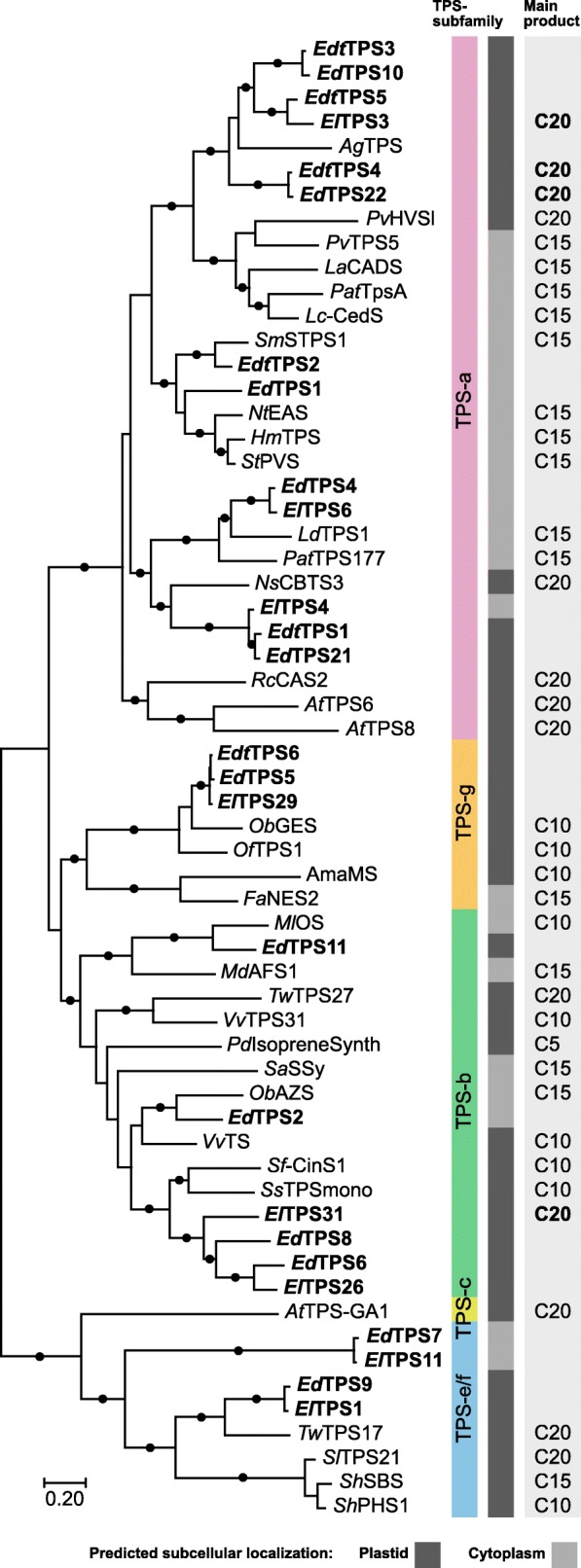
Phylogenetic analysis of *Eremophila* TPSs. Maximum likelihood tree of TPSs based on aligned protein sequences calculated using MEGA 7 [56]. Tree is drawn to scale, with branch lengths representing the number of substitutions per site. Filled circles on branches indicate bootstrap support of above 75% based on 1000 repetitions. Genbank accession numbers are listed in Tables S3 and S4 (Additional file 1). Subcellular localisation predicted using DeepLoc-1.0 [57]

Transcripts encoding candidate TPSs were identified in the libraries using homology-based searches with known TPSs. Of the identified candidates, 6, 12 and 8 likely full length TPSs could be cloned from *E. denticulata* subsp. *trisulcata*, *E. drummondii* and *E. lucida* trichome cDNA, respectively (Additional file 1: Table S3). Phylogenetic analysis of the candidate TPSs indicated notable expansions in both the TPS-a and TPS-b subfamilies (Fig. 2; Additional file 1: Table S4). Six TPSs were found to form a cluster within the TPS-a clade. The most closely related characterised TPSs to this cluster are Lamiaceae sesquiterpene synthases (sesquiTPSs) making cyclic sesquiterpenes and *Pv*HVS, a recently reported diTPS from *Prunella vulgaris* [35]. Unlike the sesquiTPSs, which were predicted to be localised to the cytosol, all of the *Eremophila* TPSs of this clade were predicted to be plastid targeted similarly to *Pv*HVS. Interestingly, this clade contained many of the most highly expressed TPSs (based on TPM) in the trichome-enriched transcriptome libraries from each species (e.g. *ElTPS3*, *EdTPS22* and *EdtTPS4*; Additional file 1: Table S3). A second *Eremophila* specific cluster was observed to group with TPSs involved in the biosynthesis of cyclic monoterpenes in the TPS-b subfamily (Fig. 2). Again, several of these candidates had high TPM values (*ElTPS31* and *EdTPS6*; Additional file 1: Table S3).

**Fig. 3:**
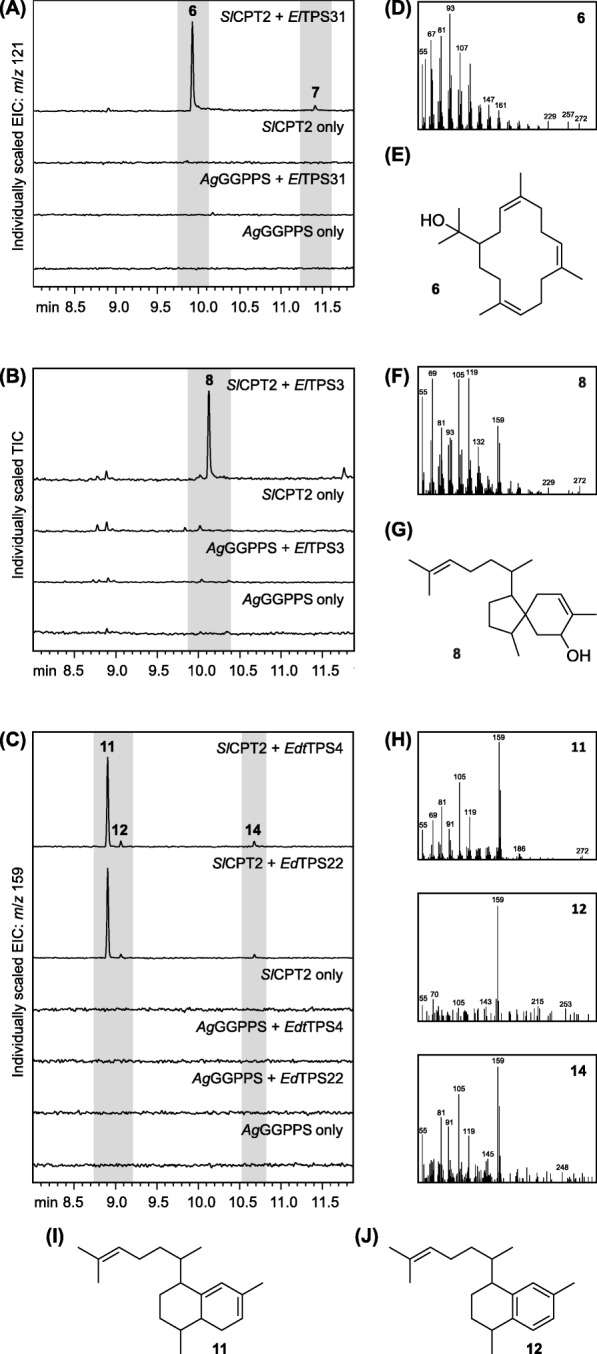
In vivo functional characterisation of *Eremophila* TPSs. **(A-C)** GC-MS chromatograms of hexane extracts of *E. coli* cultures expressing *ElTPS31*, *ElTPS3*, *EdtTPS4* and *EdTPS22* in combination with either a GGPP synthase (*AgGGPPS*) or a NNPP synthase (*SlCPT2*). **d**, **f** and **h** Mass spectra of major TPS products. **e**, **g**, **i** and **j** Chemical structures of (3*Z*,7Z,11*Z*)-cembratrien-15-ol (**6**), 5-hydroxyviscidane (**8**), 8,9-dihydroserrulat-14-ene (**11**) and serrulat-14-ene (**12**)

All 26 TPS candidates were screened for diTPS activity by *Agrobacterium*-mediated transient expression in *Nicotiana benthamiana* [58, 59]. Each TPS was transiently expressed in combinations with either a GGPPS from *Coleus forskohlii* (*CfGGPPS*) [60] or an NNPPS from *Solanum lycopersicum* (*SlCPT2*) [50]. Gas chromatography-mass spectrometry (GC-MS) analysis of leaf extracts did not show any diterpene products for any of the *Eremophila* TPSs when co-expressed with *CfGGPPS*. In contrast, co-expression of either one of *ElTPS3*, *ElTPS31*, *EdtTPS4* or *EdTPS22* with NNPP-forming *SlCPT2* resulted in diterpene production. *El*TPS31 produced **6** as a main constituent along with minor amounts of a later eluting product (**7**), while *El*TPS3 produced **8** as a main product along with minor products **9** and **10** (Additional file 3: Figure S2). *Edt*TPS4 and *Ed*TPS22 were found to have identical product profiles appearing as two major constituents, **11** and **12**, along with several minor products (Additional file 4: Figure S3). The candidates with diTPS activity were subsequently cloned into the pet28b + vector, with putative plastid transit sequences removed, for functional testing in *E. coli*. Co-expression of *ElTPS3*, *ElTPS31*, *EdtTPS4* and *EdTPS22* with *SlCPT2* resulted in the same major diterpene products as observed in *N. benthamiana* (Fig. 3). The product profiles of *El*TPS3 and *Edt*TPS4/*Ed*TPS22 when expressed in *E. coli* were, however, somewhat simplified with only trace levels of **9** and **10** detected in strains expressing *ElTPS3* and a large peak intensity shift towards **11** in strains expressing *EdtTPS4* or *EdTPS22*.

Each of the major products from *El*TPS31, *El*TPS3 and *Edt*TPS4/*Ed*TPS22 was purified from *E. coli* cultures and their structures elucidated by NMR spectroscopic analysis. The major diterpene products of *El*TPS31 and *El*TPS3 were identified as (3*Z*,7*Z*,11*Z*)-cembratrien-15-ol (**6**; Additional file 1: Table S5) and 5-hydroxyviscidane (**8**; Additional file 1: Table S6), respectively (Fig. 3). The *Z*-configuration of each of the double bonds in **6** was established by NOE correlations between the olefinic protons and the methyl groups, showing them to be on the same side (*Z* = zusammen = *cis*) of the double bond. Similarly, NOE correlations were observed between the two methylene groups attached to the double bonds, further supporting the *Z*-configuration of all double bonds. During the purification of the *Edt*TPS4/*Ed*TPS22 products, in which a reverse-phase thin-layer chromatography (RP-TLC) strategy was employed, conversion of **11** to **12** was observed by GC-MS analysis. NMR analysis of the resulting sample identified **12** as serrulat-14-ene (Additional file 1: Table S7). GC-QTOF-MS analysis of an extract of *E. coli* cultures expressing *EdtTPS4* gave an [M + H]^+^ ion of *m*/*z* 273.2569 (calc. *m*/*z* 273.2577, 4.4 ppm difference) for peak **11**, suggesting a molecular formula of C_20_H_32_ indicating that **12** (with molecular formula of C_20_H_30_) likely arises from the aromatization of **11**. Development of an alternative purification strategy using solid-phase extraction enabled the isolation of enough **11** for NMR analysis, which was identified as 8,9-dihydroserrulat-14-ene (Additional file 1: Table S8).

**Fig. 4:**
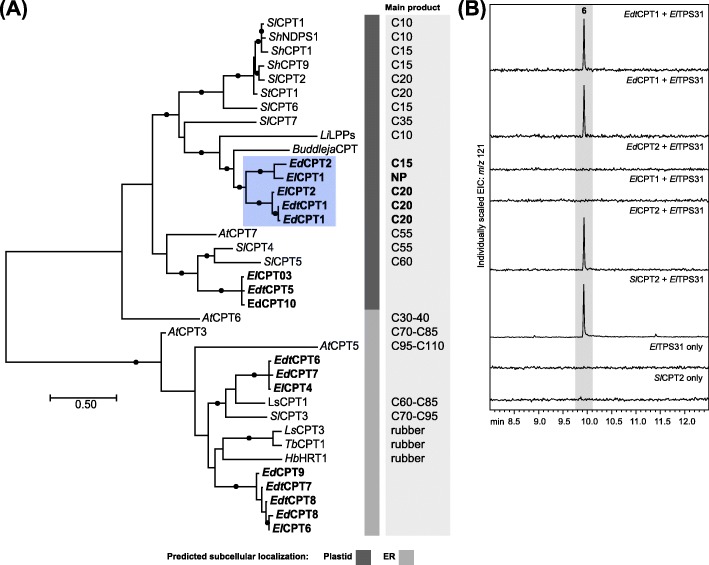
**a** Phylogenetic analysis of *Eremophila* CPTs. Maximum likelihood tree of CPTs based on aligned protein sequences calculated using MEGA 7 [56]. Tree is drawn to scale, with branch lengths representing the number of substitutions per site. Filled circles on branches indicate bootstrap support above 75% based on 1000 repetitions. Genbank accession numbers are listed in Tables S3 and S9 (Additional file 1). *Eremophila* CPTs in blue box were functionally characterised. NP = no main product detected. Subcellular localisation predicted using DeepLoc-1.0 [57]. **b** In vivo functional characterisation of *Eremophila* CPTs. GC-MS chromatograms of hexane extracts of *E. coli* cultures expressing *Eremophila CPTs* in combination with *ElTPS31*

In light of the finding that the major diterpene backbones of these species are derived from NNPP we searched the transcriptome databases for NNPPS candidates belonging to the CPT family. A family of CPTs was identified in all three species with between five and six full-length representatives in each transcriptome (Additional file 1: Table S3). A phylogenetic analysis was carried out to investigate how the *Eremophila* CPTs are related to characterised angiosperm CPTs (Fig. 4; Additional file 1: Table S9). Overall, the phylogenetic analysis showed the CPTs split in two main clusters: those localised in the plastids and those in the endoplasmic reticulum (ER). Each of these groups was further divided into two sub-clusters. For the ER localised candidates, one cluster was positioned within a clade containing CPTs involved in rubber biosynthesis [46, 48, 61] and the second cluster was associated with a clade containing long-chain polyprenyl diphosphate synthases that participate in dolichol biosynthesis [48, 62]. Of those *Eremophila* CPTs predicted to have plastid transit peptides, one group segregated with CPTs of medium and long-chain synthesising enzymes involved in plastid localised polyprenol biosynthesis [49, 63] while the other group clustered with short-chain synthesising enzymes that are involved in mono-, sesqui- and diterpene biosynthesis in *Solanum* spp. and *Lavandula* × *intermedia* [32, 39, 40, 44, 64]. Based on the phylogenetic ties with characterised short-chain CPTs, and the predicted plastid localisation along with high TPM values for three of the candidates (*EdCPT1*, *EdtCPT1* and *ElCPT2*; Additional file 1: Table S3) relative to the other CPT candidates, we hypothesized that  candidates in this latter cluster could be involved in diterpene biosynthesis in *Eremophila*.

All five candidate CPTs in this cluster were cloned from trichome RNA derived cDNA and tested for NNPP synthesising activity in *E. coli*. Cell cultures expressing the *Eremophila* *CPT*s (truncated to remove putative plastid transit sequences) were lysed and treated with alkaline phosphatase to hydrolyse the diphosphate products to their respective alcohols, extracted with hexane and analysed by GC-MS. *Ed*CPT1, *Edt*CPT1 and *El*CPT2 were found to produce NNPP as their main product and *Ed*CPT2 produced (*Z*,*Z*)-FPP as the main product (Table 1; Additional file 5: Figure S4) while *El*CPT1 produced only trace amounts of NNPP. The *CPT*s were also co-expressed with the newly established NNPP acceptor, *ElTPS31.* In support of the above results, *Ed*CPT1, *Edt*CPT1 and *El*CPT2 could all combine with *El*TPS31 to produce **6** (Fig. 4), whereas no products were observed for combinations with *Ed*CPT2 and *El*CPT1.

**Table 1:** In vivo functional characterisation of *Eremophila* CPTs. Products were detected by GC-MS as prenyl alcohols after alkaline phosphatase treatment of lysed *E. coli* cultures

	*Ed*CPT1	*Ed*CPT2	*Edt*CPT1	*El*CPT1	*El*CPT2
Main product	NNPP	(*Z*,*Z*)-FPP	NNPP	–	NNPP
Minor product	(*Z*,*Z*)-FPP	NNPP	(*Z*,*Z*)-FPP	NNPP	(*Z*,*Z*)-FPP

Leaf extracts from all three *Eremophila* species were analysed by GC-MS and examined for the presence of the TPS products. Apart from a small signal in *E. lucida* extracts corresponding to **6** (Additional file 6: Figure S5), none of the other TPS products were detected in the plant extracts.

## Discussion

### Involvement of trichomes in the biosynthesis of diterpenoids in *Eremophila*

*Eremophila* species are a rich source of novel terpenoids, particularly diterpenoids [22]. Early studies have suggested that in many *Eremophila* species diterpenoids are biosynthesised in glandular trichomes from where they are released to form part of the viscid resins that coat the surface of leaves [53, 65–67]. The three *Eremophila* species investigated in this study are all characterised by the presence of such a resin as well as short peltate glandular trichomes positioned below the layer of resin (Fig. 1). Analysis of leaf surface extracts indicated the resin from each species contained the diterpenoids of interest (Additional file 2: Figure S1). Furthermore, analysis of the trichome-enriched transcriptomes indicated a high level of activity of the MEP pathway based on TPM values (Additional file 1: Table S2), which is generally known to be involved in diterpenoid biosynthesis [68]. Taken together with the identification of the presently reported diTPSs and CPTs, it is likely that the trichomes are the site of diterpenoid biosynthesis in the *Eremophila* species examined here. This is in agreement with the known role of glandular trichomes in specialised terpenoid biosynthesis in plants [69–71].

### Serrulatane, viscidane and cembrane-type diterpenoids in *Eremophila* spp. are biosynthesised from the non-canonical terpene precursor, NNPP, by class I type terpene synthases

The majority of diterpenes found in plants are of the labdane-type and derived from the *transoid* precursor, GGPP. They are biosynthesised via a step-wise process involving the sequential action of class II and class I diTPSs typically from the subfamilies TPS-c and TPS-e/f, respectively [72]. In contrast, each of the major diterpene backbones focused on in this study was found to be biosynthesised from the *cis*-prenyl diphosphate, NNPP, by the action of a single class I TPS. Based on phylogenetic analyses, the 8,9-dihydroserrulat-14-ene synthases (*Edt*TPS4 and *Ed*TPS22) and 5-hydroxyviscidane synthase (*El*TSP3) belong to the TPS-a subfamily, whereas the (3*Z*,7*Z*,11*Z*)-cembratrien-15-ol synthase (*El*TPS31) belongs to the TPS-b subfamily (Fig. 2). Although dominated by sesquiTPSs (TPS-a) and monoterpene synthases (monoTPSs; TPS-b), a few diTPSs have been reported from these subfamilies. For example, macrocyclic diTPSs from multiple plant families form a cluster in the TPS-a subfamily and include macrocyclic diTPSs from the Euphorbiaceae [29, 73–75] and a group of root expressed diTPSs from *Arabidopsis* [30, 33]. Interestingly, the *Eremophila* diTPSs in this sub-family are not closely related to these diTPSs but appear to be more closely related to sesquiTPSs and the recently identified diTPS from *Prunella vulgaris*, *Pv*HVS (Fig. 2) [35]. Similarly, *El*TPS31 is more closely related to monoTPSs (Fig. 2) rather than the only other two diTPSs known from the TPS-b subfamily: a pair of orthologous miltiradiene synthases from *Tripterygium* spp., which accept the bicyclic class II TPS product (+)-copalyl diphosphate [76, 77]. The phylogenetic relationships of the *Eremophila* diTPSs, suggest these enzymes evolved from sesquiTPS and monoTPS progenitors, with re-acquisition of a plastid targeting sequence in the case of the TPS-a subfamily members. Such a scenario has been suggested for *Pv*HVS [35] and other TPSs where there is evidence for recent changes in substrate specificity, often accompanied by gain or loss of a functional plastid targeting sequence (for example: [78–80]).

There are few other reports of TPSs that use *cis*-prenyl diphosphates as their natural substrates *in planta* [32, 34, 39–44]. Those that are known, including the presently identified NNPP accepting TPSs from *Eremophila*, also do not cluster together in the phylogenetic tree but are scattered amongst TPSs accepting GGPP, (*E*,*E*)-FPP or GPP. Thus, it appears the ability of TPSs to accept NNPP and other *cis*-prenyl diphosphates has evolved independently multiple times in the TPS family. Indeed, in vitro testing of TPSs with *cis*-prenyl diphosphates has revealed some latent ability to accept these substrates, even when there is no evidence for them being the natural substrates *in planta* [35, 41, 80, 81]. This suggests that a barrier to the more widespread occurrence of *cis*-prenyl diphosphate derived terpenoids in plants may be the limited occurrence of short-chain CPTs rather than an inherent inability of TPSs to accept these substrates. Analysis of the terpene biosynthetic gene cluster on chromosome 8 in *Solanum* spp. provides some insight into how *cis*-prenyl diphosphate-based terpenoid metabolism could evolve through a process of co-evolution of CPTs and TPSs [32, 41, 43, 50]. This cluster contains CPTs with specialised functions as NPP, (*Z*,*Z*)-FPP and NNPP synthases. They are present in the gene cluster along with *cis*-substrate accepting TPSs with mono-, sesqui- and diterpene synthase activity which have evolved from a common TPS-e/f diterpene synthase. It is conceivable that the appearance of a CPT with short-chain synthesising activity could combine with TPSs with at least partial activity towards the new substrate to provide the initial genetic starting material for the subsequent gene duplications and specialisation of both CPTs and TPSs as observed in *Solanum*. This process would be facilitated by the commonly observed substrate promiscuity of TPSs (as reviewed by: [82]; see also [60, 81, 83, 84]), and by the ease with which TPSs can gain new functionalities with few amino acid changes (for example: [85–87]).

### Proposed reaction pathways catalysed by *El*TPS3 and *Ed*TPS22/*Edt*TPS4

The viscidane and serrulatane backbones are diterpene analogues of acoradiene and cadalane type sesquiterpenes, respectively. Reaction pathways leading to these sesquiterpene backbones are based on studies of TPSs which use (*E*,*E*)-FPP as their natural substrate but they may still be informative for proposing the reaction pathways of *El*TPS3 and *Ed*TPS22/*Edt*TPS4. The acoradiene backbone is formed from (*E,E*)-FPP via the bisabolyl cation which results from a 1,6-ring closure following an initial *trans*-*cis* isomerization of the C2-C3 bond of the (*E*,*E*)-farnesyl cation to the (*Z*,*E*)-farnesyl cation via the neutral intermediate nerolidyl diphosphate [88, 89]. After a 1,2-hydride shift (from position C6 to C7), the resulting homobisabolyl cation is transformed into the acorenyl cation in a 6,10-ring closure. Subsequent proton elimination from the isopropyl tail and formation of the C11-C12 double bond terminates the reaction. The same mechanism may be used to explain the initial steps in the biosynthesis of 5-hydroxyviscidane catalysed by *El*TPS3, but without the need of the *trans*-*cis* isomerization due to the *cis*-configuration of the three stereogenic double bonds of the substrate nerylneryl diphosphate. Thus, after the 1,6-ring closure and a 1,2-hydride shift from C6 to C7 (Additional file 7: Intermediate IIa in Figure S6), a 6,10-ring closure would afford the viscidanyl cation. The reaction is proposed to be terminated by water quenching of the carbocation at C5 after a 1,5-hydride shift from C5 to C11 (Additional file 7: Figure S6).

The cadalane type backbones can be derived from two routes that involve 1,10-cyclization [90, 91]. In the first route, ionization of (*E*,*E*)-FPP is followed by direct 1,10-cyclization to yield a (*E*,*E*)-germacradienyl cation. In the second route, cyclization is preceded by the previously described *trans*-*cis* isomerization pathway resulting in the formation of a (*Z*,*E*)-germacradienyl cation. Following further rearrangements of the two intermediates the pathways converge at the cadinenyl cation after 1,6-ring closure [90, 91]. Subsequent hydride shifts followed by proton elimination then lead to a range of cadalane type sesquiterpenes [90]. An alternative route follows the same pathway to the bisabolyl cation as reported for acoradiene type sesquiterpene biosynthesis [91]. From the bisabolyl cation further rearrangement and a second ring closure yields the cadinenyl cation. The reaction pathway leading from NNPP to 8,9-dihydroserrulat-14-ene catalysed by *Ed*TPS22/*Edt*TPS4 is potentially more likely to follow the latter route given the stereochemistry of the starting substrate (Additional file 7: Figure S6). Thus, without the need of the *trans*-*cis* isomerization of the activated nerylneryl cation, 1,6-ring closure to form intermediate I would be followed by a 1,3 hydride shift from C5 to C7 (Additional file 7: Intermediate IIb in Figure S6), which after a 5,10-ring closure would form the serrulatanyl cation. A 1,4 hydride shift from C4 to C11 and enzyme catalysed proton abstraction from C5 would then lead to 8,9-dihydroserrulat-14-ene.

### Evolution and function of diterpenoids in *Eremophila*

A broader examination of the diterpenoids isolated from *Eremophila* spp. indicates that, based on structural similarities, the pathway described here—a *cis*-prenyl diphosphate precursor cyclized directly by class I type TPSs—is common to other species in this genus [15, 17, 19, 22]. Along with the widespread occurrence of viscidane, serrulatane and cembrane-type diterpenoids, examples from diverse *Eremophila* spp. exist of linear and bisabolene-type diterpenoids with *cis*-configured double bonds, which suggests they are also derived from NNPP [92, 93]. Significantly, other genera in Myoporeae and the sister tribe Leucophylleae contain species with serrulatane type diterpenoids [20, 94] making it likely that a similar biosynthetic pathway is present in these genera as well. Furthermore, searches for homologous sequences in publicly available transcriptome databases (1KP database [95];) of other Scrophulariaceae species identified a TPS with a putative plastid transit peptide from *Anticharis glandulosa* (*Ag*TPS, tribe Aptosimeae [96];), that clusters within the subclade of diterpenoid associated TPS-a enzymes from *Eremophila* (Fig. 2), and a putative short-chain CPT from *Buddleja* sp. (*Buddleja*CPT, tribe Buddlejeae [96];), also predicted to be plastid localised and clustering with *Eremophila* NNPP producing CPTs (Fig. 4). Taken together, these data suggest that the alternative biosynthetic route to diterpenoids via NNPP may have arisen before the divergence of these separate lineages in Scrophulariaceae.

To date, *Eremophila* (and potentially related genera as described above) is the only known example of a lineage of plants that has evolved such extensive diterpenoid chemistry derived largely from the alternative *cisoid* precursor, NNPP. The broadened chemical diversity resulting from the use of this alternative substrate may offer particular selective advantages, which could explain the remarkable abundance and diversity of these unusual diterpenoids across the entire genus. However, the biological functions of the diterpenoids found in *Eremophila* species remain uncharacterised. The viscid resin of which they are a part is thought to be an adaptation to aridity mediated by its ability to reduce water loss by increasing resistance to transpiration and by lowering leaf temperature by increasing reflectance of sunlight [65, 97]. It is also likely that the resin and the diterpenoids therein are involved in defence against herbivores and pathogens. Although again there is no data available relating to this aspect of *Eremophila* biology, in vitro studies on the bioactivity of serrulatanes show a broad range of antimicrobial activities [12–14, 16, 17, 20]. Adding credence to the idea that serrulatanes may have an antimicrobial function *in planta*, bees have been reported to collect serrulatane-containing resin from the leaves of the closely related species, *Myoporum insulare,* for making bee-glue, an antiseptic material used by bees to seal their hives [94].

## Conclusions

Our study has identified a biosynthetic route to three of the major diterpene backbones found in *Eremophila* species. The identified CPTs and TPSs are the starting points of biosynthetic networks involving multiple enzyme-catalysed steps which lead to the more complex and bioactive diterpenoids characteristic of species in this genus, many of which show promise as new drugs or drug leads. The finding that trichomes are the likely site of biosynthesis of diterpenoids and development of trichome-enriched transcriptome databases is providing  valuable knowledge and resources that can be used to identify downstream terpenoid biosynthetic enzymes, [98, 99].

In this work we show that NNPP is the precursor for all three types of diterpenoids investigated here. The broad distribution of these and similar compounds across *Eremophila* and related genera suggests this alternative pathway to specialised diterpenoids is common across the plant lineage. With its species richness and broad geographic distribution across Australia, *Eremophila* thus provides an eminent model system for the study of the evolution of terpenoid chemical diversity.

## Materials and methods

### Plant material and glandular trichome RNA isolation

Plant material was harvested from plants growing in the greenhouse at the University of Copenhagen (Frederiksberg, Denmark) under natural light supplemented with growth lights during winter months (7 am – 7 pm) with an average day/night temperature of 18 °*C. Eremophila lucida* and *E. drummondii* material was sourced as described in Tahtah et al. [18] and Wubshet et al. [19], respectively. The *E. denticulata* subsp. *trisulcata* specimen was sourced from the State Flora Nursery of South Australia. Voucher specimens of *E. lucida* (UCPH-PLEN-AH4), *E. drummondii* (UCPH-PLEN-AH3) and *E. denticulata* subsp. *trisulcata* (﻿UCPH-PLEN-AH6) have been deposited at Herbarium C, National History Museum, University of Copenhagen.

To isolate glandular trichomes from *Eremophila* spp. a novel trichome isolation method based on surface contact freezing was developed. Fresh leaves were tightly sandwiched between two plastic plates and frozen on dry ice. The plastic plates with leaves were exposed to room temperature for 10 s and then abruptly opened leaving trichomes and resin with minimal other leaf material attached to the plates. The plastic plates were washed down with 1.5 mL pre-chilled lysis-buffer (RNAqueous-Micro Total RNA Isolation Kit, Thermo Fisher Scientific) supplemented with 1:10 Plant RNA Isolation Aid (Thermo Fisher Scientific) and 300 mg PVP40. The lysis solution was collected into 2 mL round bottom Eppendorf tubes containing glass beads of different sizes (1.5 mm, 1 mm and 0.5 mm diameter) and subjected to a cell disruption step (3 cycles of 3 min at 3000 rpm in a TissueLyser II, QIAGEN, Hilden, Germany), with cooling of samples on dry ice for 2 min between cycles. The lysed trichome samples were centrifuged at 20000 g for 10 min. The supernatant was transferred to a binding column provided with the RNAqueous-Micro Total RNA Isolation Kit. Total RNA was isolated following standard kit protocol conditions and with on-column DNA digestion with supplied DNase I. RNA integrity and concentration was determined using the RNA-nano assay on the Agilent 2100 Bioanalyzer (Agilent Technologies, Santa Clara, CA, USA).

### Transcriptome analysis

RNA-seq libraries were generated with TruSeq Stranded mRNA LT Sample Prep Kit (Illumina San Diego, USA) using poly-A selection. Library preparation and sequencing was conducted by Macrogen (Seoul, South Korea) with paired ends (2 × 101 bp) on a HiSeq 2500 (Illumina), according to the manufacturer’s instructions. Transcriptome assembly was carried out by Sequentia Biotech SL. A quality check was performed on the raw sequencing data using BBDuk (https://sourceforge.net/projects/bbmap/), where minimum read length was set to 35 bp and the Phred quality score to 35. The high quality reads were used as input to perform transcriptome assembly after normalization (with Trinity v2.1.1) [100]. Quality control and filtering of the transcriptome assembly was conducted in three steps. First, the expression levels of all the transcripts were quantified with the software Kallisto [101] and then all transcripts with no expression levels were removed. Second, to reduce the redundancy of the dataset, for each gene only the isoform encoding the longest protein was retained; for the non-coding transcripts, the longest sequence was kept. Finally, all the transcripts having a match with a non-plant organism were filtered out. To obtain the expression quantification of the assembled transcripts in the three samples, the trimmed reads were processed with the software Kallisto and TPM (Transcripts Per Million) values were calculated for all the transcripts. A summary of the transcriptomic data is given in (Additional file 1: Table S2). In addition, de novo transcriptomes were generated with the RNAseq assembly tool of the CLC genomic assembly software (version 11, QIAGEN) using default settings.

The putative function of assembled transcripts as encoding CPTs or TPSs was inferred using two approaches. First, a BLAST based homolog search was applied on the generated transcriptome libraries using massblast (https://github.com/averissimo/mass-blast). Secondly, transcripts were scanned for PFAM domains with HMMER (version 3.1b1), using HMM models for the N-terminal (Acc PF01397.20) and C-terminal (Acc PF03936.15) part of terpene synthases (Pfam-A v29). Eventually, the open reading frame-prediction tool of the CLC Main Workbench (version 8.0.1, QIAGEN) was used to identify coding sequences. Phylogenetic analyses were carried out using MEGA 7 [56] as described in Heskes et al. [102].

For the MVA and MEP pathway analysis annotated *Arabidopsis thaliana* genes of the MEP and MVA pathways were selected from the NCBI Protein database and used to query the *Eremophila* trichome transcriptomes using tBLASTn with an E-value cutoff of 1E-10. All BLAST hits were checked for open reading frames of at least 200 amino acids and translated into protein sequences. BLAST hits with a minimum overall identity and query coverage of 50% were selected.

### Microscopic analysis of leaf cross sections

Fresh leaf material was embedded in 5% agarose and sectioned in 20 μm sections using a HM 650 V vibrating microtome (Microm International, Walldorf, Germany). Sections were mounted in water for imaging using a DMI 4000B inverted microscope (Leica Microsystems, Wetzler, Germany). True color imaging was done under bright field at 20 × magnification. Microscopic images were taken by Leica software and further processed using ImageJ (version 1.51j8).

### Functional characterisation of biosynthetic candidate genes in *N. benthamiana*

To characterise the function of putative CPTs and TPSs from *Eremophila*, cloned candidates were tested using *Agrobacterium*-mediated transient expression in *Nicotiana benthamiana*. Generation of cDNA from isolated leaf RNA for each *Eremophila* spp. was done using the iScript cDNA Synthesis Kit (Biorad, Hercules, CA). Gene specific primers (Additional file 1: Table S10) were designed with USER overhangs to amplify full length coding sequences of candidates from generated *Eremophila* cDNA libraries. A DNA fragment of the coding sequence of *SlCPT2* [50] was obtained by commercial synthesis (Thermo Fisher Scientific). Where candidates were not predicted to be full length in the transcriptomic databases transcripts were elongated to full length using homologous sequences found within the three generated *Eremophila* transcriptomes. For transient expression in *N. benthamiana*, amplicons of coding sequences were integrated into the pCAMBIA130035Su vector by USER cloning [103]. Competent *Agrobacterium* AGL-1 cells were transformed with plasmid DNA and T-DNA encoded target genes were transiently co-expressed in 4–6 week old *N. benthamiana* plants [58, 59] together with the gene silencing suppressor p19 [104] and the gene encoding the *C. forskohlii* enzyme, 1-deoxy-d-xylulose 5-phosphate synthase (*Cf*DXS) [60]. Six days post-infiltration, two leaf discs (3 cm diameter) from individual leaves were excised and extracted in 1 mL hexane at room temperature for 1 h on an orbital shaker at 220 rpm. Plant material was collected by centrifugation and the organic phase was transferred to GC vials for GC-MS analysis.

### GC-MS analysis

Samples were analysed by GC-MS using a Shimadzu GCMS-QP2010 Ultra (Shimadzu, Kyoto, Japan) fitted with an HP-5MS UI column (20 m × 0.18 mm i.d., 0.25 μm film thickness; Agilent) using H_2_ as the carrier gas. The injection port was operated in splitless mode with a starting temperature of 40 °C which was held for 1 min and then ramped to 250 °C over 4 min. The GC oven program was as follows: 60 °C for 1 min, ramp to 150 °C at 30 °C min^− 1^, ramp to 250 °C at 15 °C min^− 1^, ramp to 290 °C at 30 °C min^− 1^, hold for 3 min. The MS used electron impact (EI) ionisation with the ion source voltage and temperature set to 70 eV and 300 °C, respectively. For analysis of *E. coli* strains expressing *Eremophila* CPTs and treated with phosphatase the oven program was started at 40 °C. Data was analysed using GCMSsolution software v4.20 (Shimadzu).

### Characterisation of *El*TPS3, *El*TPS31, *Edt*TPS4 and *Ed*TPS22 in *E. coli* and isolation of 6, 8, 11 and 12

Coding sequences of N-terminally truncated *Eremophila* TPSs (*El*TPS3Δ1-23, *El*TPS31Δ1-54, *Edt*TPS4Δ1-59 and *Ed*TPS22Δ1-59) were cloned into the pet28b + expression vector). Each TPS construct was used to co-transform *E. coli* EXPRESS BL21 competent cells (Lucigen, Middleton, WI) along with pIRS [105] and pACYCDuet vector carrying either *Abies grandis* *GGPP* synthase [106] or N-terminally truncated *Solanum lycopersicum* *NNPP* synthase (*SlCPT2*) [50]. 2 mL cultures were grown at 37 °C until OD_600_ 0.8 was reached, cooled to 16 °C, and induced with the addition of IPTG (final concentration 1 mM). The cultures were then grown at 18 °C for 48 h at 200 rpm, centrifuged and 1 mL aliquots of supernatant were extracted with 0.3 mL hexane at room temperature for 1 h on an orbital shaker at 220 rpm. The resulting hexane extracts were analysed by GC-MS as for *N. benthamiana* samples. For compound purification, 200 mL cultures were grown in unbaffled 2 L conical flasks with the above-described conditions. Large-scale cultures were centrifuged and the supernatant extracted with an equal volume of hexane twice. The hexane extracts were reduced in volume by rotary evaporation and the concentrate fractionated using RP-TLC with methanol as the mobile phase to give **6**, **8** and **12**. To purify **11**, the concentrated hexane extract was applied onto a Dual layer florisil/Na_2_SO_4_ SPE cartridge (6 mL, Supelco, PA, USA) and eluted with 1% ethyl acetate in hexane.

To obtain accurate mass data on the TPS products, extracts of *E. coli* cultures expressing *ElTPS3, ElTPS31, EdtTPS4* and *EdTPS22* with *SlCPT2* were analysed by GC-QTOF-MS using a Scion 456-GC coupled to a MicroTOF II MS equipped with an APCI source (Bruker Daltonik, Bremen, Germany). Samples were injected in splitless mode with an injection port temperature of 250 °C. The GC was fitted with a 30 m BR-5 ms column (5% phenyl, 95% dimethyl arylene siloxane; Bruker) with 250 μm ID and 0.25 μm film thickness. The carrier gas was H_2_ with a constant linear velocity of 30 cm s^− 1^. The oven program was as follows: initial temperature of 60 °C held for 1 min, followed by a linear ramp to 130 °C at 20 °C min^− 1^, which was then ramped to 250 °C at 4 °C min^− 1^. Finally the oven was ramped to 290 °C at 30 °C min^− 1^ and held for 4 min. The APCI source was operated in positive ionization mode with the following settings: capillary voltage, 3000 V; corona discharge needle, 2000 nA; nebulizer gas pressure, 3 bar; dry gas flow, 2.5 L min^− 1^; dry gas temperature, 250 °C. A mass range of 50 to 700 *m*/*z* was used.

### Characterisation of *Eremophila* CPTs in *E. coli*

To test for the ability of the selected *Eremophila* CPTs to produce NNPP, the same *E. coli* system as employed for TPS characterisation was used. Coding sequences of N-terminally truncated *Eremophila* CPTs (*Ed*CPT1Δ1-58, *Ed*CPT2Δ1-58, *Edt*CPT1Δ1-58, *El*CPT1Δ1-60 and *El*CPT2Δ1-58) were cloned into pACYC-Duet vector and used to co-transform *E. coli* EXPRESS BL21 competent cells (Lucigen, Middleton, WI) with pIRS [105] and either empty pet28b + or pet28b+:*ElTPS31*. Culture conditions were the same as for TPS characterisation except that 15 mL cultures of the strains not expressing *El*TPS31 were grown. These cultures were subjected to a cell lysis procedure using a cell disruptor (Constant Systems Ltd., Northants, UK) set to 25 kpsi. The lysate was adjusted to 20 mL with water and centrifuged 8000 g for 20 min. 10 mL of supernatant was treated with 20 μL calf intestinal phosphatase (New England Biolabs, Ipswich, MA) and incubated overnight at 37 °C. Samples were then extracted twice with 1 mL hexane. Hexane extracts were combined and concentrated to 50 uL under a stream of N_2_ and analysed by GC-MS. Strains expressing *ElTSP31* were prepared and analysed as for TPS characterisation.

### Metabolite analysis of *Eremophila* spp.

For GC-MS analysis of *Eremophila* spp. freshly harvested leaves were ground under liquid N_2_, extracted in hexane while shaking for 1 h at 24 °C. Hexane samples were analysed by GC-MS as per *E. coli* and *N. benthamiana* samples. For LC-HRMS analysis freshly harvested leaves were dipped for 15 s in 100% ethyl acetate. The extracts were dried for 1 h in a speedvac centrifuge and resuspended in 80% acetonitrile. Acetonitrile extracts were analysed using an Ultimate 3000 UHPLC+ Focused system (Dionex Corporation, Sunnyvale, CA) coupled to a Bruker Compact ESI-QTOF-MS (Bruker) system. Samples were separated on a Kinetex XB-C18 column (100 × 2.1 mm ID, 1.7 μm particle size, 100 Å pore size; Phenomenex Inc., Torrance, CA) maintained at 40 °C with a flow rate of 0.3 mL min^− 1^ and mobile phase consisting of 0.05% (v/v) formic acid in water (solvent A) and 0.05% (v/v) formic acid in acetonitrile (solvent B). The LC method was as follows: 0–1 min, 10% B; 1–23 min, 10–100% B; 23–25 min, 100%; 25–25.5 min, 100–10%; 25.5–30.5 min, 10% B. Mass spectra were acquired in positive ion mode with the following ESI settings: capillary voltage, 4000 V; end plate offset, − 500 V; dry gas temperature, 220 °C; dry gas flow of 8 L min^− 1^; nebulizer pressure, 2 bar. Data were analysed using DataAnalysis 4.1 (Bruker).

### NMR spectroscopy

Nuclear magnetic resonance spectroscopy (NMR) experiments were recorded in CDCl_3_ on a 600 MHz Bruker Avance III instrument at a proton frequency of 600.13 MHz, using a 1.7 mm cryogenically cooled TCI probehead. All NMR experiments were performed in automation (temperature equilibration to 300 K, optimization of lock parameters, gradient shimming, and setting of receiver gain) using IconNMR ver 4.2 (Bruker Biospin, Karlsruhe, Germany). ^1^H NMR spectra were acquired with 30°-pulses and 64 k data points. Standard 2D homo- and heteronuclear experiments were acquired with 2048 or 1730 (HSQC) data points in the direct dimension and 512 (DQF-COSY) or 256 (multiplicity-edited HSQC and HMBC) data points in the indirect dimension. Topspin ver. 3.5 (Bruker Biospin) was used for acquisition and processing of NMR data.

## Supplementary information

**Additional file 1: Table S1** Transcriptome sequencing statistics. **Table S2.** MVA and MEP pathway analysis. **Table S3.** List of candidate terpene synthases (TPSs) and *cis*-prenyl transferases (CPTs) studied in this work. *E. lucida* = *El*, *E. drummondii* = *Ed* and *E. denticulata* subsp. *trisulcata* = *Edt.* DeepLoc-1.0 was used to predict protein subcellular localisation [57]. **Table S4.** Protein sequences used in the terpene synthase phylogenetic analysis. **Table S5.** ^1^H (600 MHz) and ^13^C (150 MHz) data of (3*Z*,7*Z*,11*Z*)-cembratrien-15-ol (**6**) in CDCl_3_. **Table S6.** ^1^H (600 MHz) and ^13^C (150 MHz) data of 5-hydroxyviscidane (**8**) in CDCl_3_. **Table S7.** ^1^H (600 MHz) and ^13^C (150 MHz) data of serrulat-14-ene (**12**) in CDCl_3_. **Table S8.** ^1^H (600 MHz) and ^13^C (150 MHz) data of 8,9-dihydroserrulat-14-ene (**11**) in CDCl_3_. **Table S9.** Protein sequences used in the *cis*-prenyl transferase phylogenetic analysis. **Table S10.** List of primers used in this study.

**Additional file 2: Figure S1.** Liquid chromatography-high-resolution mass spectrometry (LC-HRMS) extracted ion chromatograms (EIC) of ethyl acetate leaf-surface wash extracts prepared from *E. lucida, E. denticulata* subsp. *trisulcata* and *E. drummondii*. Depicted EICs represent the combined *m*/*z* values of the calculated [M + H]^+^ ions (± 0.05) of known diterpenoids reported from these species. *E. lucida*: 321.2424 [18]; *E. denticulata* subsp. *trisulcata*: 333.2066, 335.2221 [52, 54]; *E. drummondii*: 333.2058, 335.2221, 363.1809, 349.2014, 351.2169, 513.2474, 527.2638, 421.2586, 435.2744, 367.2121, 409.2226, 451.2332, 365.1964 [19, 54]. Predicted molecular formulas are within ±2.0 ppm error of calculated values.

**Additional file 3: Figure S2.** *In planta* functional characterisation of *El*TPS31 and *El*TPS3. **(A)** and **(B)** GC-MS chromatograms of hexane extracts of *N. benthamiana* leaves transiently expressing *ElTPS31* and *ElTPS3* in combination with either GGPP synthase (*CfGGPPS*) or NNPP synthase (*SlCPT2*). **(C)** Mass spectra of TPS products.

**Additional file 4: Figure S3.** *In planta* functional characterisation of *Edt*TPS4 and *Ed*TPS22. **(A)** GC-MS chromatograms of hexane extracts of *N. benthamiana* leaves transiently expressing *EdtTPS4* and *EdTPS22* in combination with either GGPP synthase (*CfGGPPS*) or NNPP synthase (*SlCPT2*). **(B)** Mass spectra of main TPS products. **(C)** Mass spectra of minor products (inset window of **A**).

**Additional file 5: Figure S4. (A)** In vivo functional characterisation of *Eremophila* CPTs. GC-MS chromatograms of hexane extracts of *E. coli* cultures treated with alkaline phosphatase. **(B)** Mass spectra of prenyl alcohols: nerylnerol (a), (2*Z*,6*Z*)-farnesol (b) and nerol (c).

**Additional file 6: Figure S5. (A)** GC-MS analysis of a hexane extract of *E. lucida* leaves. Only **6**, the product of *El*TPS31, was detected in the plant. Comparison trace is of an extract of *N. benthamiana* leaves transiently expressing *ElTPS31* with NNPPS (*SlCPT2*). **(B)** Mass spectrum of **6** produced by heterologous expression in *N. benthamiana* compared to *E. lucida* leaf extract.

**Additional file 7: Figure S6.** Proposed reaction pathways catalysed by *Ed*TPS22/*Edt*TPS4 to 8,9-dihydroserrulat-14-ene and *El*TPS3 to 5-hydroxyviscidane from nerylneryl diphosphate.

## Data Availability

The RNA-seq data generated during the current study have been submitted to the Sequence Read Archive (SRA) at NCBI with the following accession number: PRJNA601673. The cDNA sequences of *Eremophila* TPSs and CPTs reported in this study are available through GenBank. Accessions numbers are listed in Table S3 (Additional file 1).

## Abbreviations

(*E*,*E*)-FPPS: (*E*,*E*)-farnesyl diphosphate synthase
CPT: *Cis*-prenyl transferases
diTPS: diterpene synthase
DMAPP: Dimethylallyl diphosphate
GC-MS: Gas chromatography-mass spectrometry
GFPPS: Geranylfarnesyl diphosphate synthase
GGPP: Geranylgeranyl diphosphate
GGPPS: Geranylgeranyl diphosphate synthase
GPP: Geranyl diphosphate
GPPS: Geranyl diphosphate synthase
IPP: Isopentenyl diphosphate
LC-HRMS: Liquid chromatography-high resolution mass spectrometry
MEP: 2-*C*-methyl-D-erythritol 4-phosphate
monoTPSs: monoterpenes synthases
MVA: Mevalonate
NMR: Nuclear magnetic resonance
NNPP: Nerylneryl diphosphate
RP-TLC: Reverse-phase thin-layer chromatography
sesquiTPS: sesquiterpene synthase
TPM: Transcripts per million
TPS: Terpene synthase
*trans*-PTs: *trans*-prenyl transferases
