# Supplementary material for: Nerylneryl diphosphate is the precursor of serrulatane, viscidane and cembrane-type diterpenoids in Eremophila species
Source: BMC Plant Biol. 2020 Feb 28;20:91. doi: 10.1186/s12870-020-2293-x (PMC7049213; doi:10.1186/s12870-020-2293-x)
Supplement: Supplementary file 1 — Additional file 1: Table S1 Transcriptome sequencing statistics. Table S2. MVA and MEP pathway analysis. Table S3. List of candidate terpene synthases (TPSs) and cis-prenyl transferases (CPTs) studied in this work. E. lucida = El, E. drummondii = Ed and E. denticulata subsp. trisulcata = Edt. DeepLoc-1.0 was used to predict protein subcellular localisation [57]. Table S4. Protein sequences used in the terpene synthase phylogenetic analysis. Table S5. 1H (600 MHz) and 13C (150 MHz) data of (3Z,7Z,11Z)-cembratrien-15-ol (6) in CDCl3. Table S6. 1H (600 MHz) and 13C (150 MHz) data of 5-hydroxyviscidane (8) in CDCl3. Table S7. 1H (600 MHz) and 13C (150 MHz) data of serrulat-14-ene (12) in CDCl3. Table S8. 1H (600 MHz) and 13C (150 MHz) data of 8,9-dihydroserrulat-14-ene (11) in CDCl3. Table S9. Protein sequences used in the cis-prenyl transferase phylogenetic analysis. Table S10. List of primers used in this study. [file 12870_2020_2293_MOESM1_ESM.pdf]

Supplementary Tables S1-S10

Title

Nerylneryl diphosphate is the precursor of serrulatane, viscidane and cembrane-type diterpenoids in *Eremophila* species.

Oliver Gericke[1,2], Nikolaj Lervad Hansen[1,2], Gustav Blichfeldt Pedersen[1,2], Louise Kjaerulff[3], Dan Luo[1,2], Dan Staerk[3], Birger Lindberg Møller[1,2], Irini Pateraki[1,2] and Allison Maree Heskes[1,2,*]

[1]Plant Biochemistry Laboratory, Department of Plant and Environmental Sciences, University of Copenhagen, Thorvaldsensvej 40, DK-1871 Frederiksberg C, Denmark,

[2]Center for Synthetic Biology "bioSYNergy", Department of Plant and Environmental Sciences, University of Copenhagen, Thorvaldsensvej 40, DK-1871 Frederiksberg C, Denmark,

[3]Department of Drug Design and Pharmacology, Faculty of Health and Medical Sciences, University of Copenhagen, DK-2100 Copenhagen, Denmark.

Table S1 Transcriptome sequencing statistics.

Parameter	*E. lucida*	*E. denticulata* subsp. *trisulcata*	*E. drummondii*
Total read bases (bp)	14599741698	12700647990	14298445568
GC raw data (%)	46.42	45.63	46.63
AT raw data (%)	53.58	54.37	53.37
Q30 raw data (%)	93.54	92.98	93.23
n raw reads	144551898	125748990	141568768
n trimmed Reads	126896626	108851044	123461628
n transcript	47787	62439	60818
Smallest transcript (bp)	200	200	200
Longest transcript (bp)	12832	15552	14850
n bases (Mbp)	38.22	43.65	44.42
mean length (bp)	800	699.11	730.4
N50 (bp)	1402	1123	1256
GC assembly (%)	43	43	44
Read back mapping (%)	88	86	85

Table S2 MVA and MEP pathway analysis**.** Annotated *Arabidopsis thaliana* genes of the MEP and MVA pathways were selected from the NCBI Protein database and used to identify homolgous genes in the *Eremophila* trichome transcriptomes using tBLASTn. All BLAST hits were checked for open reading frames of at least 200 amino acids and translated into protein sequences. BLAST hits with a minimum overall identity and query coverage of 50% were selected. n = number of homologs identified in the transcriptome, TPM = Transcripts Per Million counts, QC = query coverage, I = percent identity, E = E-value.

Enzyme	Query Accession No.	*E. lucida*					*E. denticulata subsp. trisulcata*					*E. drummondii*				
		n	TPM	QC	I	E	n	TPM	QC	I	E	n	TPM	QC	I	E
DXS	Q38854.2	3	1650	91	73.5	0	3	1311	92	71.9	0	3	2847	91	72.3	0
DXR	Q9XFS9.2	1	1242	56	67.4	1E-72	1	735	64	66.0	2E-126	1	1851	88	73.7	1E-153
MCT	P69834.1	1	201	73	81.9	2E-129	1	178	73	82.3	5E-130	1	210	73	81.9	3E-130
CMK	NP_180261.1	1	424	73	79.2	7E-145	1	460	46	80.6	1E-86	1	336	63	78.3	6E-116
MDS	Q9CAK8.1	2	1406	70	90.1	7E-104	1	728	92	74.6	7E-109	1	40	71	91.4	2E-106
HDS	NP_001332136.1	1	863	92	82.8	0	1	242	37	87.9	1E-153	1	2466	93	81.8	0
HDR	NP_567965.1	2	1574	89	82.1	0	2	3739	100	76.8	0	2	2135	100	81.4	0
IDI	Q38929.3	2	824	79	86.1	5E-147	1	115	78	88.1	9E-150	1	945	78	88.1	8E-149
GPPS	NP_001031483.1; NP_850234.1	2	378	100	76	1E-162	1	255	55	59.8	3E-79	1	291	100	76.0	7E-162
GGPPS	P34802.2; O04046.2	3	925	81	74	1E-152	2	506	81	74.1	9E-147	2	114	81	77.7	8E-157
FPPS	Q09152.2; Q43315.1	2	921	99	79.1	0	2	157	99	78.6	0	2	288	99	77.1	0
AACT	AAM00280.1	1	291	99	85.8	0	3	584	99	85.7	0	3	967	99	85.3	0
HMGS	P54873.2	1	388	99	82.9	0	1	971	99	82.0	0	1	299	99	81.8	0
HMGR	P14891.1; P43256.1	3	263	64	86.3	0	3	769	92	71.7	0	3	287	56	85.5	0
MVK	NP_001190411.1	1	765	100	69.1	4E-168	1	27	91	67.2	2E-116	1	614	100	68.6	2E-168
PMK	Q9C6T1.1	1	376	100	70.7	0	1	255	57	67.4	3E-115	1	312	100	70.1	0
MVD	O23722.1	1	376	99	73.5	0	1	450	99	73.2	0	1	600	99	72.6	0

Table S3 List of candidate terpene synthases (TPSs) and *cis*-prenyl transferases (CPTs) studied in this work. *E. lucida* = *El*, *E. drummondii* = *Ed* and *E. denticulata* subsp. *trisulcata* = *Edt.* DeepLoc-1.0 was used to predict protein subcellular localisation [1].

Name	Species	Accession No.	DeepLoc-1.0 localisation	TPM
**Terpene synthase (TPS)**				
EdtTPS1	*Eremophila denticulata* subsp. *trisulcata*	MN958352	Plastid	8
EdtTPS2	*Eremophila denticulata* subsp. *trisulcata*	MN958353	Cytoplasm	3
EdtTPS3	*Eremophila denticulata* subsp. *trisulcata*	MN958354	Plastid	29
EdtTPS4	*Eremophila denticulata* subsp. *trisulcata*	MN958355	Plastid	724
EdtTPS5	*Eremophila denticulata* subsp. *trisulcata*	MN958356	Plastid	446
EdtTPS6	*Eremophila denticulata* subsp. *trisulcata*	MN958357	Plastid	28
EdTPS1	*Eremophila drummondii*	MN958358	Cytoplasm	4
EdTPS2	*Eremophila drummondii*	MN958359	Cytoplasm	14
EdTPS4	*Eremophila drummondii*	MN958360	Cytoplasm	223
EdTPS5	*Eremophila drummondii*	MN958361	Plastid	3
EdTPS6	*Eremophila drummondii*	MN958362	Plastid	1500
EdTPS7	*Eremophila drummondii*	MN958363	Cytoplasm	6
EdTPS8	*Eremophila drummondii*	MN958364	Plastid	477
EdTPS9	*Eremophila drummondii*	MN958365	Plastid	2
EdTPS10	*Eremophila drummondii*	MN958366	Plastid	700
EdTPS11	*Eremophila drummondii*	MN958367	Plastid	41
EdTPS21	*Eremophila drummondii*	MN958368	Plastid	52
EdTPS22	*Eremophila drummondii*	MN958369	Plastid	1500
ElTPS1	*Eremophila lucida*	MN958370	Plastid	2
ElTPS3	*Eremophila lucida*	MN958371	Plastid	1563
ElTPS4	*Eremophila lucida*	MN958372	Cytoplasm	364
ElTPS6	*Eremophila lucida*	MN958373	Cytoplasm	36
ElTPS11	*Eremophila lucida*	MN958374	Cytoplasm	1
ElTPS26	*Eremophila lucida*	MN958375	Plastid	156

ElTPS29	*Eremophila lucida*	MN958376	Plastid	11
ElTPS31	*Eremophila lucida*	MN958377	Plastid	1228
***cis*-Prenyl transferase (CPT)**				
EdtCPT1	*Eremophila denticulata* subsp. *trisulcata*	MN958378	Plastid	2020
EdtCPT5	*Eremophila denticulata* subsp. *trisulcata*	MN958379	Plastid	3
EdtCPT6	*Eremophila denticulata* subsp. *trisulcata*	MN958380	ER	5
EdtCPT7	*Eremophila denticulata* subsp. *trisulcata*	MN958381	ER	11
EdtCPT8	*Eremophila denticulata* subsp. *trisulcata*	MN958382	ER	10
EdCPT1	*Eremophila drummondii*	MN958383	Plastid	2215
EdCPT2	*Eremophila drummondii*	MN958384	Plastid	133
EdCPT7	*Eremophila drummondii*	MN958385	ER	11
EdCPT8	*Eremophila drummondii*	MN958386	ER	8
EdCPT9	*Eremophila drummondii*	MN958387	ER	33
EdCPT10	*Eremophila drummondii*	MN958388	Plastid	1
ElCPT1	*Eremophila lucida*	MN958389	Plastid	8
ElCPT2	*Eremophila lucida*	MN958390	Plastid	4814
ElCPT3	*Eremophila lucida*	MN958391	Plastid	3
ElCPT4	*Eremophila lucida*	MN958392	ER	4
ElCPT6	*Eremophila lucida*	MN958393	ER	35

Table S4 Protein sequences used in the terpene synthase phylogenetic analysis. DeepLoc-1.0 was used to predict protein subcellular localisation [1].

Protein	Species	Family	Main product	DeepLoc-1.0 predicted localization	Genbank accession No.
*Ag*TPS	*Anticharis glandulosa*	Scrophulariaceae	Unknown	Plastid	1KP-EJBY-2044143
*Ama*MS	*Antirrhinum majus*	Plantaginaceae	Monoterpene	Plastid	AAO41727.1
*At*TPS-GA1	*Arabidopsis thaliana*	Brassicaceae	Diterpene-diphos.	Plastid	AAA53632.1
*At*TPS6	*Arabidopsis thaliana*	Brassicaceae	Diterpene	Plastid	Q84UU9.2
*At*TPS8	*Arabidopsis thaliana*	Brassicaceae	Diterpene	Plastid	NP_193754.2
*Fa*NES2	*Fragaria* x *ananassa*	Rosaceae	Sesquiterpene	Cytoplasm	CAD57081.1
*Hm*TPS	*Hyoscyamus muticus*	Solanaceae	Sesquiterpene	Cytoplasm	AAA86337.1
*La*CADS	*Lavandula angustifolia*	Lamiaceae	Sesquiterpene	Cytoplasm	AGL98418.1
*Ld*TPS1	*Phyla dulcis*	Verbenaceae	Sesquiterpene	Cytoplasm	JQ731632.1
*Md*AFS1	*Malus domestica*	Rosaceae	Sesquiterpene	Plastid	AAO22848
*Ml*OS	*Mimulus lewisii*	Phrymaceae	Monoterpene	Cytoplasm	KF857262.1
*Ns*CBTS3	*Nicotiana sylvestris*	Solanaceae	Diterpene	Plastid	ADI87448.1
*Nt*EAS	*Nicotiana attenuata*	Solanaceae	Sesquiterpene	Cytoplasm	AAP05760.1
*Ob*AZS	*Ocimum basilicum*	Lamiaceae	Sesquiterpene	Cytoplasm	AAV63788.1
*Ob*GES	*Ocimum basilicum*	Lamiaceae	Monoterpene	Plastid	AAR11765.1
*Of*TPS1	*Osmanthus fragrans*	Oleaceae	Monoterpene	Plastid	KT591180.1
*Pat*TPS177	*Pogostemon cablin*	Lamiaceae	Sesquiterpene	Cytoplasm	AY508730.1
*Pd*IsopreneSynth	*Populus deltoides*	Salicaceae	Isoprene	Plastid	AEK70966.1
*Pv*HVSl	*Prunella vulgaris*	Lamiaceae	Diterpene	Plastid	MH926015.1
*Rc*CAS2	*Ricinus communis*	Euphorbiaceae	Diterpene	Plastid	XM_002513288.3
*Sa*SSy	*Santalum album*	Santalaceae	Sesquiterpene	Plastid	HQ343276
Sf-CinS1	*Salvia fruticosa*	Lamiaceae	Monoterpene	Plastid	ABH07677.1
*Sm*STPS1	*Salvia miltiorrhiza*	Lamiaceae	Sesquiterpene	Cytoplasm	ARM19967.1
*Ss*TPSmono	*Salvia stenophylla*	Lamiaceae	Monoterpene	Plastid	AAM89254.1
*Tw*TPS17	*Tripterygium wilfordii*	Celastraceae	Diterpene	Plastid	KU948695
*Tw*TPS27	*Tripterygium wilfordii*	Celastraceae	Diterpene	Plastid	KU948697
*Vv*TPS31	*Vitis vinifera*	Vitaceae	Monoterpene	Plastid	ADR74209.1

*Vv*TS	*Vitis vinifera*	Vitaceae	Monoterpene	Plastid	AAS79351.1
Lc-CedS	Leucosceptrum canum	Lamiaceae	Sesquiterpene	Cytoplasm	QBP05430
*Pat*TpsA	*Pogostemon cablin*	Lamiaceae	Sesquiterpene	Cytoplasm	AAS86319
*Pv*TPS5	*Prunella vulgaris*	Lamiaceae	Sesquiterpene	Cytoplasm	MH926017
*Sh*PHS1	*Solanum habrochaites*	Solanaceae	Monoterpene	Plastid	JN990689
*Sh*SBS	*Solanum habrochaites*	Solanaceae	Sesquiterpene	Plastid	FJ194970.1
*Sl*TPS21	*Solanum lycopersicum*	Solanaceae	Diterpene	Plastid	AGK82800.1
*St*PVS	*Solanum tuberosum*	Solanaceae	Sesquiterpene	Cytoplasm	BAA82108

Table S5 ^{1}H (600 MHz) and ^{13}C (150 MHz) data of (3*Z*,7*Z*,11*Z*)-cembratrien-15-ol (**6**) in $CDCl_3$.

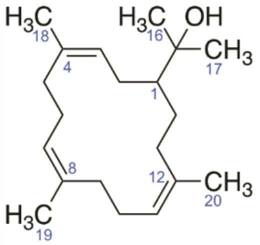

Pos.	δ_C[a]	δ_H (nH, coupling patterns, coupling constants)[b,c]	HMBC correlations
1	50.3	1.26 (1H, m)	15
2	30.5	A: 2.06 (1H, ddd, 14.5 Hz, 9.5 Hz, 7.7 Hz)	1, 3, 4, 15
		B: 2.22 (1H, br d. 14.5 Hz)	1, 3, 4, 15
3	125.7	5.23 (1H, ddq, 9.5 Hz, 5.1 Hz, 1.0 Hz)	2, 5, 18
4	135.9	-	
5	28.2	2.13 (1H, q, 7.4 Hz)	3, 4, 6
6	32.2	A: 1.92 (1H, m)	5, 7, 8, 19
		B: 2.30 (1H, dt, 13.1 Hz, 6.7 Hz)	5, 7, 8, 19
7	125.5	5.17 (1H, tq, 7.9 Hz, 1.0 Hz)	6, 9, 19
8	135.9	-	
9	33.2	A: 1.89 (1H, m)	7, 8, 10
		B:1.99 (1H, m)	7, 8, 10
10	28.9	1.99 (1H, m)	11, 12
11	125.3	5.18 (1H, t, 7.1 Hz)	10, 13, 20
12	135.9	-	
13	32.1	A: 1.76 (1H, m)	11, 12, 14
		B: 2.39 (1H, m)	11, 12, 14
14	30.8	A: 1.04 (1H, m)	1
		B: 1.79 (1H, m)	1, 13
15	73.9	-	
16	27.2	1.20 (3H, s)	1, 15, 17
17	27.3	1.21 (3H, s)	1, 15, 16
18	24.2	1.70 (3H, s)	2, 3, 4
19	24.2	1.70 (3H, s)	6, 7, 8
20	24.2	1.70 (3H, s)	10, 11, 12

[a]referenced to solvent peak at 77.16, [b]referenced to residual solvent peak at 7.26, [c]coupling patterns reported as apparent splitting patterns with the following abbreviations: s = singlet, d = doublet, t = triplet, sext = sextet, br = broad, m = unresolved multiplet (due to overlap).

Table S6 ^{1}H (600 MHz) and ^{13}C (150 MHz) data of 5-hydroxyviscidane (**8**) in $CDCl_3$.

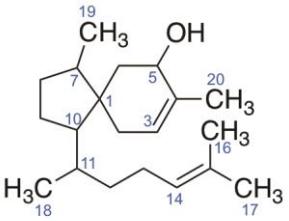

Pos.	δ_C[a]	δ_H (nH, coupling patterns, coupling constants)[b,c]	HMBC correlations
1	47.7	-	
2	30.4	A: 1.63 (1H, dd, 17.3 Hz, 3.0 Hz)<br>B: 2.06 (1H, m)	1, 3, 4, 6
3	123.6	5.41 (1H, br d, 3.0 Hz)	4, 5, 20
4	135.8	-	
5	69.5	4.15 (1H, br s)	4
6	40.6	A: 1.73 (1H, dd, 12.6 Hz, 10.0 Hz)<br>B: 1.87 (1H, m)	1, 5, 7
7	39.5	1.89 (1H, m)	9, 19
8	29.9	A: 1.15 (1H, m)<br>B: 1.79 (1H, m)	1, 7, 9, 10, 19
9	26.6	A: 1.34 (1H, m)<br>B: 1.88 (1H, m)	8, 10, 11
10	50.4	1.56 (1H, m)	1, 2, 6, 12
11	33.9	1.43 (1H, m)	10, 13, 18
12	36.2	A: 1.06 (1H, dddd, 14.3 Hz, 9.6 Hz, 9.6 Hz, 4.7 Hz)<br>B: 1.43 (1H, m)	10, 11, 14, 18
13	25.6	A: 1.87 (1H, m)<br>B: 2.04 (1H, m)	12, 14, 15
14	125.2	5.09 (1H, br t, 7.1 Hz)	16, 17
15	131.3	-	
16	25.9	1.68 (3H, s)	14, 15, 17
17	17.8	1.60 (3H, s)	14, 15, 16
18	20.2	0.96 (3H, d, 6.6 Hz)	10, 11, 12
19	17.6	0.88 (3H, d, 7.1 Hz)	1, 7, 8
20	19.1	1.76 (3H, s)	3, 4, 5

[a]referenced to solvent peak at 77.16, [b]referenced to residual solvent peak at 7.26, [c]coupling patterns reported as apparent splitting patterns with the following abbreviations: s = singlet, d = doublet, t = triplet, br = broad, m = unresolved multiplet (due to overlap).

Table S7 ^{1}H (600 MHz) and ^{13}C (150 MHz) data of serrulat-14-ene (**12**) in $CDCl_3$.

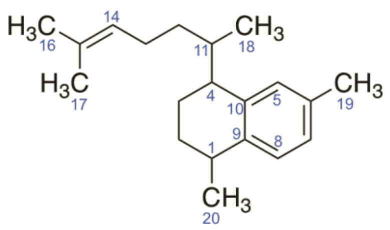

Pos.	δ_C[a]	δ_H (nH, coupling patterns, coupling constants)[b,c]	HMBC correlations	ROESY correlations
1	33.8	2.71 (1H, m)	2, 9	
2	32.3	A: 1.29 (1H, m)		
		B: 1.95 (1H, m)		20
3	23.2	A: 1.54 (1H, m)		18
		B: 1.89 (1H, m)	1, 10	
4	44.9	2.74 (1H, m)	2, 3, 10, 11, 12	5, 18
5	129.2	6.99 (1H, br s)	4, 7, 9, 19	4, 11, 18, 19
6	135.2	-		
7	126.9	6.90 (1H, br d, 7.9 Hz)	5, 9, 19	19
8	124.4	7.09 (1H, d, 7.9 Hz)	1, 6, 10	20
9	141.0	-		
10	140.3	-		
11	37.5	2.08 (1H, m)		5
12	32.6	A: 1.03 (1H, m)		
		B: 1.11 (1H, m)	11, 13, 18	
13	27.0	A: 1.82 (1H, m)	12, 14, 15	
		B: 1.95 (1H, m)	12, 14, 15	
14	125.6	4.96 (1H, m)	16, 17	
15	131.8	-		
16	17.4	1.52 (3H, br s)	14, 15, 17	
17	25.7	1.64 (3H, br s)	14, 15, 16	
18	18.3	1.01 (3H, d, 6.9 Hz)	4, 11, 12	3A, 4, 5
19	20.9	2.26 (3H, s)	5, 6, 7	5, 7
20	22.3	1.24 (3H, d, 6.9 Hz)	1, 2, 9	2B, 8

[a]referenced to solvent peak at 77.16, [b]referenced to residual solvent peak at 7.26, [c]coupling patterns reported as apparent splitting patterns with the following abbreviations: s = singlet, d = doublet, br = broad, m = unresolved multiplet (due to overlap).

Table S8. ^{1}H (600 MHz) and ^{13}C (150 MHz) data of 8,9-dihydroserrulat-14-ene (**11**) in $CDCl_3$.

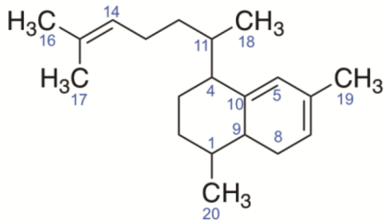

Pos.	δ_C[a]	δ_H (nH, coupling patterns, coupling constants)[b,c,d]	HMBC correlations
1	39.0	1.66 (1H, m)	
2	29.4	n.d.	
3	25.6	A: 1.71 (1H, m)<br>B: n.d.	2
4	50.2	1.77 (1H, m)	
5	122.5	5.48 (1H, br s)	4, 7, 9, 19
6	129.1	-	
7	116.7	5.11 (1H, br s)	
8	28.3	A: 1.98 (1H, m)<br>B: 2.59 (1H, m)	1, 6, 10
9	35.0	2.56 (1H, m)	1, 7
10	141.8	-	
11	30.7	1.79 (1H, m)	
12	34.9	A: 1.56 (1H, m)<br>B: 1.03 (1H, m)	
13	25.4	A: 2.07 (1H, m)<br>B: 1.91 (1H, m)	 12, 14, 15
14	125.0	5.12 (1H, br t, 7.7 Hz)	13
15	131.1	-	
16	17.6	1.62 (3H, br s)	14, 15, 17
17	25.6	1.69 (3H, br s)	14, 15, 16
18	18.1	0.83 (3H, d, 6.5 Hz)	4, 11, 12
19	21.7	1.63 (3H, m)	5, 6, 7
20	12.6	0.95 (3H, d, 7.0 Hz)	1, 2, 9

[a]referenced to solvent peak at 77.16, [b]referenced to residual solvent peak at 7.26, [c]coupling patterns reported as apparent splitting patterns with the following abbreviations: s = singlet, d = doublet, t = triplet, br = broad, m = unresolved multiplet (due to overlap), [d]n.d. = not determined.

Table S9 Protein sequences used in the *cis*-prenyl transferase phylogenetic analysis. DeepLoc-1.0 was used to predict protein subcellular localisation [1].

**Protein**	**Species**	**Family**	**Main product chain length**	**DeepLoc-1.0 predicted localisation**	**Genbank accession No.**
*At*CPT3	*Arabidopsis thaliana*	Brassicaceae	C95-C110	ER	NM_127311.5
*At*CPT5	*Arabidopsis thaliana*	Brassicaceae	C70-C85	ER	NP_200858
*At*CPT6	*Arabidopsis thaliana*	Brassicaceae	C30-40	ER	NP_568882
*At*CPT7	*Arabidopsis thaliana*	Brassicaceae	C55	Plastid	NP_200685
*Buddleja*CPT	*Buddleja* sp.	Scrophulariaceae	Unknown	Plastid	1KP-GRFT_2073126
*Hb*HRT1	*Hevea brasiliensis.*	Euphorbiaceae	Rubber	ER	BAB71776
*Li*LPPs	*Lavandula* x *intermedia*	Lamiaceae	C10	Plastid	JX985358.1
*Ls*CPT1	*Lactuca sativa*	Asteraceae	C60-C85	ER	KF752486
*Ls*CPT3	*Lactuca sativa*	Asteraceae	Rubber	ER	KF752488
*Sh*CPT1	*Solanum habrochaites*	Solanaceae	C15	Plastid	AGK82828.1
*Sh*CPT9	*Solanum habrochaites*	Solanaceae	C15	Plastid	KC808001.1
*Sh*NDPS1	*Solanum habrochaites*	Solanaceae	C10	Plastid	KF494864.1
*Sl*CPT1	*Solanum lycopersicum*	Solanaceae	C10	Plastid	NM_001247704.1
*Sl*CPT2	*Solanum lycopersicum*	Solanaceae	C20	Plastid	JX943884
*Sl*CPT3	*Solanum lycopersicum*	Solanaceae	C65	ER	JX943885.1
*Sl*CPT4	*Solanum lycopersicum*	Solanaceae	C55	Plastid	JX943886
*Sl*CPT5	*Solanum lycopersicum*	Solanaceae	C60	Plastid	JX943887.1
*Sl*CPT6	*Solanum lycopersicum*	Solanaceae	C15	Plastid	JX943888.1
*Sl*CPT7	*Solanum lycopersicum*	Solanaceae	C35	Plastid	JX943889
*St*CPT1	*Solanum tuberosum*	Solanaceae	C20	Plastid	XM_006361518.2
*Tb*CPT1	*Taraxacum brevicorniculatum*	Asteraceae	Rubber	ER	JQ991925

Table S10 List of primers used in this study.

Target	Primer	Sequence
Cloning of full length Eremophila TPS genes into pCAMBIA130035Su for Agrobacterium-mediated transient expression in N. benthamiana		
EdTPS1	412	GGCTTAAUATGGCCTCAACAAATTATGAAAACGTAGT
EdTPS1	422	GGTTTAAUTTAGACGTTAACAGAGTCCACGAGCAACGCTG
EdTPS2	72F	GGCTTAAUATGGAGGCAAGAAGGTGC
EdTPS2	72R	GGTTTAAUTTATGGCAGATCGAAAGGTTC
EdTPS4	71F	GGCTTAAUATGGACCTGAATACCAAAGTG
EdTPS4	71R	GGTTTAAUTTAATCTTCTGAAACAGGATCG
EdTPS5	84F	GGCTTAAUATGGCCTGTATGAATAGCACC
EdTPS5	205	GGTTTAAUTTAGACAGCAAGGGGAGTAAAGAAGAG
EdTPS6	66F	GGCTTAAUATGGCGGCAATGGTAACG
EdTPS6	66R	GGTTTAAUCTACAGAATAGGCTCAAACAACAAGC
EdTPS7	70F	GGCTTAAUATGGATTCCTCATCAGTACTGTTATC
EdTPS7	70R	GGTTTAAUTCAAGAAATTAAGGGCTCCAAATG
EdTPS8	63F	GGCTTAAUATGGCTGCAATGACAATCCATAC
EdTPS8	63R	GGTTTAAUTTATTCGATAGGCTCAAACAACAATC
EdTPS9	406	GGCTTAAUATGTCTCTTCAATTCTCCATTAATTCCAC
EdTPS9	82R	GGTTTAAUTCAAAGTTCATTCAAAACGATAGGC
EdTPS10	57F	GGCTTAAUATGGCTGCTTTCATCAGCG
EdTPS10	57R	GGTTTAAUTTAAATGTCGATGGGATCCAC
EdTPS11	49F	GGCTTAAUATGCACCAGTTAGTAGGATCTTC
EdTPS11	73R	GGTTTAAUTCA AAC AAC GGG AAG AGG C
EdTPS21	61F	GGCTTAAUATGCGGTCTAAAGCCTCAG
EdTPS21	61R	GGTTTAAUTCAAATTGCCAGCACTGG
EdTPS22	60F	GGCTTAAUATGGCTGCCGCTCCC
EdTPS22	60R	GGTTTAAUTTAAATGTCGATTGGCTTCAGAAATAG
EdtTPS1	61F	GGCTTAAUATGCGGTCTAAAGCCTCAG
EdtTPS1	419	GGTTTAAUTTAAATTGCCAGTACTGGTTCAATGAGCACGG
EdtTPS2	56F	GGCTTAAUATGGCTTCAACAACTGTTG
EdtTPS2	56R	GGTTTAAUTCAGATCTTAATGGGATCGACG
EdtTPS3	57F	GGCTTAAUATGGCTGCTTTCATCAGCG
EdtTPS3	57R	GGTTTAAUTTAAATGTCGATGGGATCCAC
EdtTPS4	60F	GGCTTAAUATGGCTGCCGCTCCC
EdtTPS4	69R	GGTTTAAUTTAAATGTCGAACGGCTTCAG
EdtTPS5	55F	GGCTTAAUATGGCTACAGTGATCAGCAG
EdtTPS5	55R	GGTTTAAUCTAAATAATGAGGGGATCGACAAG
EdtTPS6	200	GGCTTAAUATGGCTTGTATGAAGAGCACCGT
EdtTPS6	205	GGTTTAAUTTAGACAGCAAGGGGAGTAAAGAAGAG
ElTPS1	406	GGCTTAAUATGTCTCTTCAATTCTCCATTAATTCCAC
ElTPS1	416	GGTTTAAUTTAAAGTTCATCCAAAACGATAGGCTCGTGAA

ElTPS3	381	GGCTTAAUATGGCTACTGTGATCAACAGC
ElTPS3	383	GGTTTAAUTTACTATATAATGATGGGATCGACAAGCAAAG
ElTPS4	409	GGCTTAAUATGGAAATTCAGTCATCAGTTTGCCCAAT
ElTPS4	419	GGTTTAAUTTAAATTGCCAGTACTGGTTCAATGAGCACGG
ElTP6	410	GGCTTAAUATGGACCAGAAATACCAAAGTGTTTTAGT
ElTP6	420	GGTTTAAUTTAATCTTCTGAAACAGGATCGATGAAAAGTT
ElTPS11	411	GGCTTAAUATGGAGTCCTCATCAGTATTGTTATCAAT
ElTPS11	421	GGTTTAAUTTAAGAAATTAAGGGCTCCAAATGTGAATGTT
ElTPS26	198	GGCTTAAUATGGGGGCAATGTTTATGCAGAG
ElTPS26	203	GGTTTAAUTTAAATTATTTTATAGAGAATAGGCTCAAACAACA
ElTPS29	200	GGCTTAAUATGGCTTGTATGAAGAGCACCGT
ElTPS29	205	GGTTTAAUTTAGACAGCAAGGGGAGTAAAGAAGAG
ElTPS31	201	GGCTTAAUATGGCTGCAATGGCAATGAATTT
ElTPS31	206	GGTTTAAUTTAAATAGAAATAGGCTTAAAGAGCAGG
EdCPT1	74F	GGCTTAAUATGCAGGTCTCACTTCAGTTTCC
EdCPT1	76R	GGTTTAAUTTATTTTATTCGTCCACCGTAACG
EdCPT2	77F	GGCTTAAUATGAATTTTTTTTGTTTCGGTCTGC
EdCPT2	80R	GGTTTAAUCTAATTTTTGCGTTCACCAAAC
EdtCPT1	74F	GGCTTAAUATGCAGGTCTCACTTCAGTTTCC
EdtCPT1	74R	GGTTTAAUTTATTTCATTCGTCCACCGTAACG
ElCPT1	267	GGCTTAAUATGAAATCTTTTTGTTTCGGGCTGC
ElCPT1	277	GGTTTAAUTTACTAATTTTTGCGTTCACCAAACTGCCTT
ElCPT2	268	GGCTTAAUATGCAGGTCTCACTTCAGTT
ElCPT2	278	GGTTTAAUTTATTATTTCTTTCGTCCACCGTAACGCCTT
SlCPT2	253	GGCTTAAUATGAACTCTTCAATAGTGTC
SlCPT2	254	GGTTTAAUTTAATATGTGTGTCCACCAAAACGTCTATGC
Cloning of N-terminally truncated genes into pet28b+ for expression in E. coli.		
EdTPS22	463	AGGAGATATACCATGGCCATGGCTGCTACCACCGCTGATCTTGA
EdTPS22	467	GGTGGTGGTGCTCGATTAAATGTCGATTGGCTTCAGAAATAGAG
EdtTPS4	465	AGGAGATATACCATGGCTGCTGCCACCGCTGATCTTGATGGTCG
EdtTPS4	469	GGTGGTGGTGCTCGATTAAATGTCGAACGGCTTCAGAAATAGAG
ElTPS3	439	AGGAGATATACCATGCAAAGTATCCTGCGTAAAGATCTTGAAAG
ElTPS3	440	GGTGGTGGTGCTCGACTATATAATGATGGGATCGACAAGCAAAG
ElTPS31	444	AGGAGATATACCATGGCTGAAGTAGAAGGCCCCATCGCCCGACG
ElTPS31	445	GGTGGTGGTGCTCGATCAAATAGAAATAGGCTTAAAGAGCAGGC
Cloning of N-terminally truncated genes into pACYCDuet MC1 for expression in E. coli.		
EdCPT1	247F	AAGGAGATATACATAATGCCTTTATCGTCTAAGTTCC
EdCPT1	247R	CTTTACCAGACTCGATTATTTTATTCGTCCACCGTAAC
EdCPT2	248F	AAGGAGATATACATAATGCCTGTACCCAATTCTTC
EdCPT2	246R	CTTTACCAGACTCGACTAATTTTTGCGTTCACCAAAC
EdtCPT1	247F	AAGGAGATATACATAATGCCTTTATCGTCTAAGTTCC
EdtCPT1	249R	CTTTACCAGACTCGATTATTTCATTCGTCCACCG
ElCPT1	537	GACTCATATGCCCAATTCTTCATGGTC

ElCPT1	538	CATACTCGAGTTACTAATTTTTGCGTTCACCAAACTGCCTT
ElCPT2	539	GACTCATATGCCTGTATCGTCTAAGTTCCGT
ElCPT2	540	CATACTCGAGTTATTATTTCTTTCGTCCACCGTAACGCCTT
SlCPT2	161	AAGGAGATATACATATGTCTGATCGTGGACTCAGC
SlCPT2	162	CTTTACCAGACTCGAGTCAATATGTGTGTCCACCAA
AgGGPPS	259	GACTCATATGTTTGACTTCAAGGAGTATC
AgGGPPS	260	CATACTCGAGTCAGTTTTGTCTGAATGCAATGTAATCT

References

1. Almagro Armenteros JJ, Sønderby CK, Sønderby SK, Nielsen H, Winther O. DeepLoc: prediction of protein subcellular localization using deep learning. Bioinformatics. 2017;33:3387–3395.
