# Supplementary figures and images for: Nerylneryl diphosphate is the precursor of serrulatane, viscidane and cembrane-type diterpenoids in Eremophila species
Source: BMC Plant Biol. 2020 Feb 28;20:91. doi: 10.1186/s12870-020-2293-x (PMC7049213; doi:10.1186/s12870-020-2293-x)

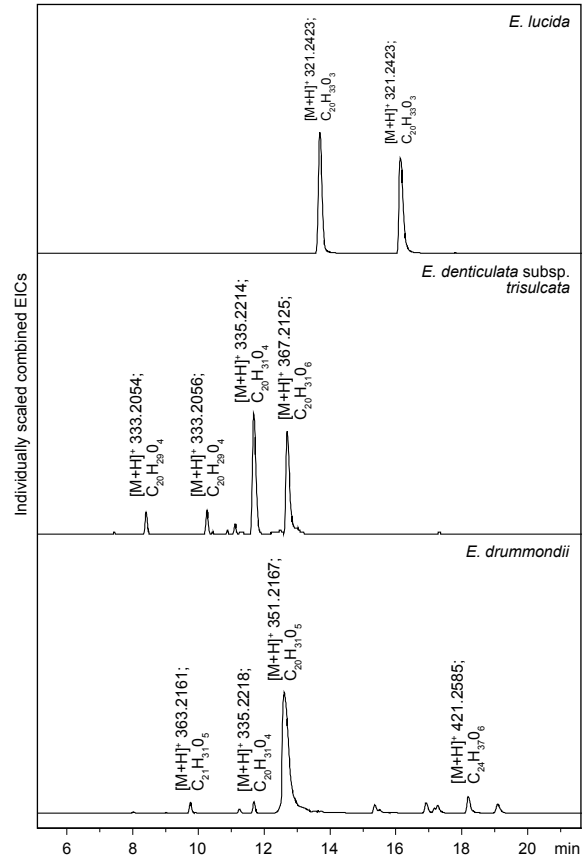

Figure S1

Supplement: Supplementary file 2 — Additional file 2: Figure S1. Liquid chromatography-high-resolution mass spectrometry (LC-HRMS) extracted ion chromatograms (EIC) of ethyl acetate leaf-surface wash extracts prepared from E. lucida, E. denticulata subsp. trisulcata and E. drummondii. Depicted EICs represent the combined m/z values of the calculated [M + H]+ ions (± 0.05) of known diterpenoids reported from these species. E. lucida: 321.2424 [18]; E. denticulata subsp. trisulcata: 333.2066, 335.2221 [52, 54]; E. drummondii: 333.2058, 335.2221, 363.1809, 349.2014, 351.2169, 513.2474, 527.2638, 421.2586, 435.2744, 367.2121, 409.2226, 451.2332, 365.1964 [19, 54]. Predicted molecular formulas are within ±2.0 ppm error of calculated values. [file 12870_2020_2293_MOESM2_ESM.pdf]

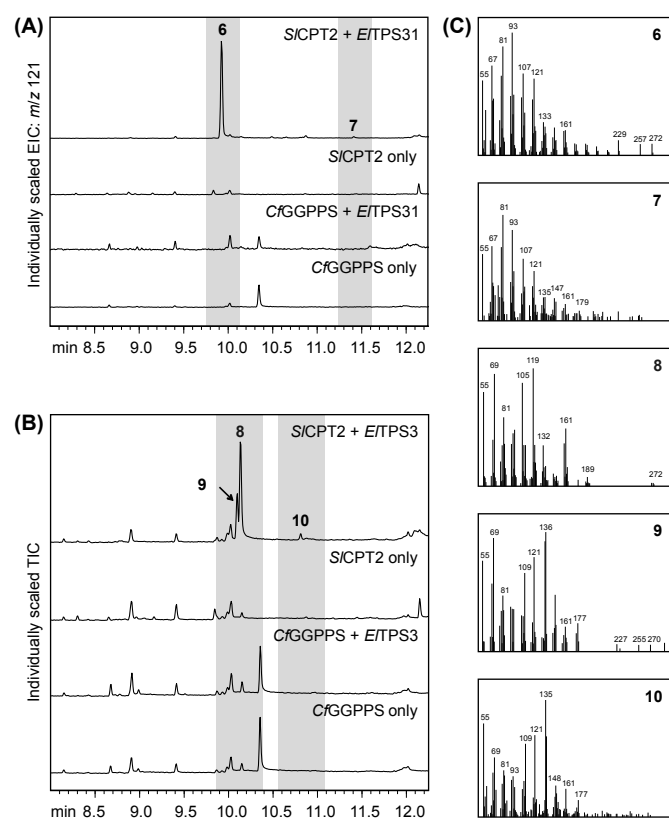

Figure S2

Supplement: Supplementary file 3 — Additional file 3: Figure S2. In planta functional characterisation of ElTPS31 and ElTPS3. (A) and (B) GC-MS chromatograms of hexane extracts of N. benthamiana leaves transiently expressing ElTPS31 and ElTPS3 in combination with either GGPP synthase (CfGGPPS) or NNPP synthase (SlCPT2). (C) Mass spectra of TPS products. [file 12870_2020_2293_MOESM3_ESM.pdf]

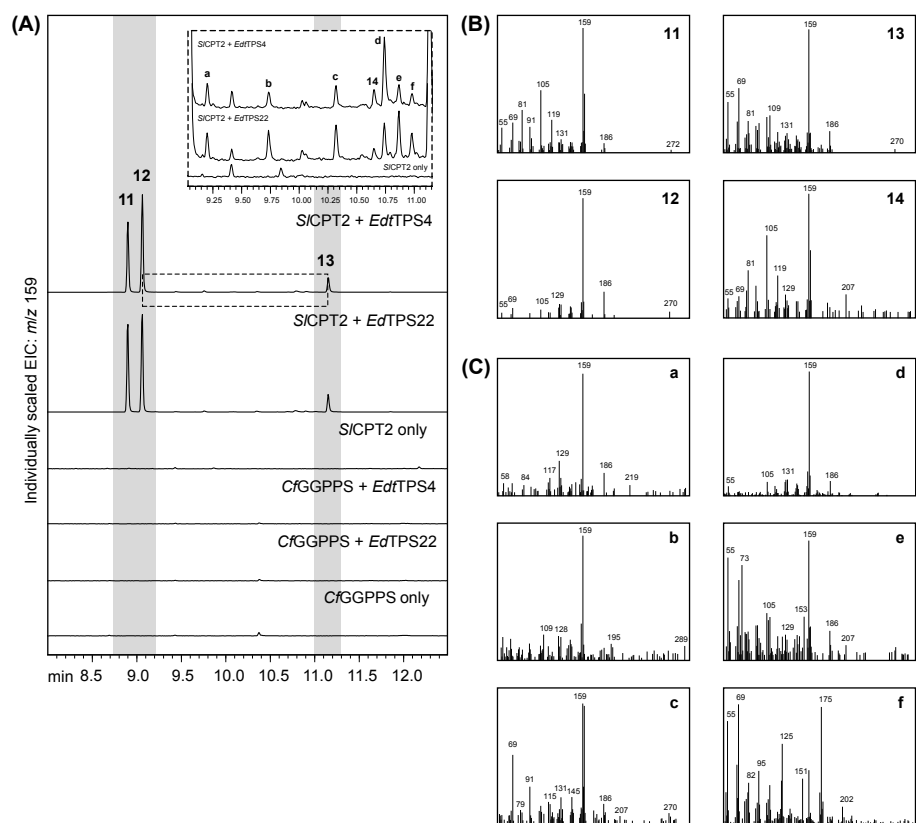

Figure S3

Supplement: Supplementary file 4 — Additional file 4: Figure S3. In planta functional characterisation of EdtTPS4 and EdTPS22. (A) GC-MS chromatograms of hexane extracts of N. benthamiana leaves transiently expressing EdtTPS4 and EdTPS22 in combination with either GGPP synthase (CfGGPPS) or NNPP synthase (SlCPT2). (B) Mass spectra of main TPS products. (C) Mass spectra of minor products (inset window of A). [file 12870_2020_2293_MOESM4_ESM.pdf]

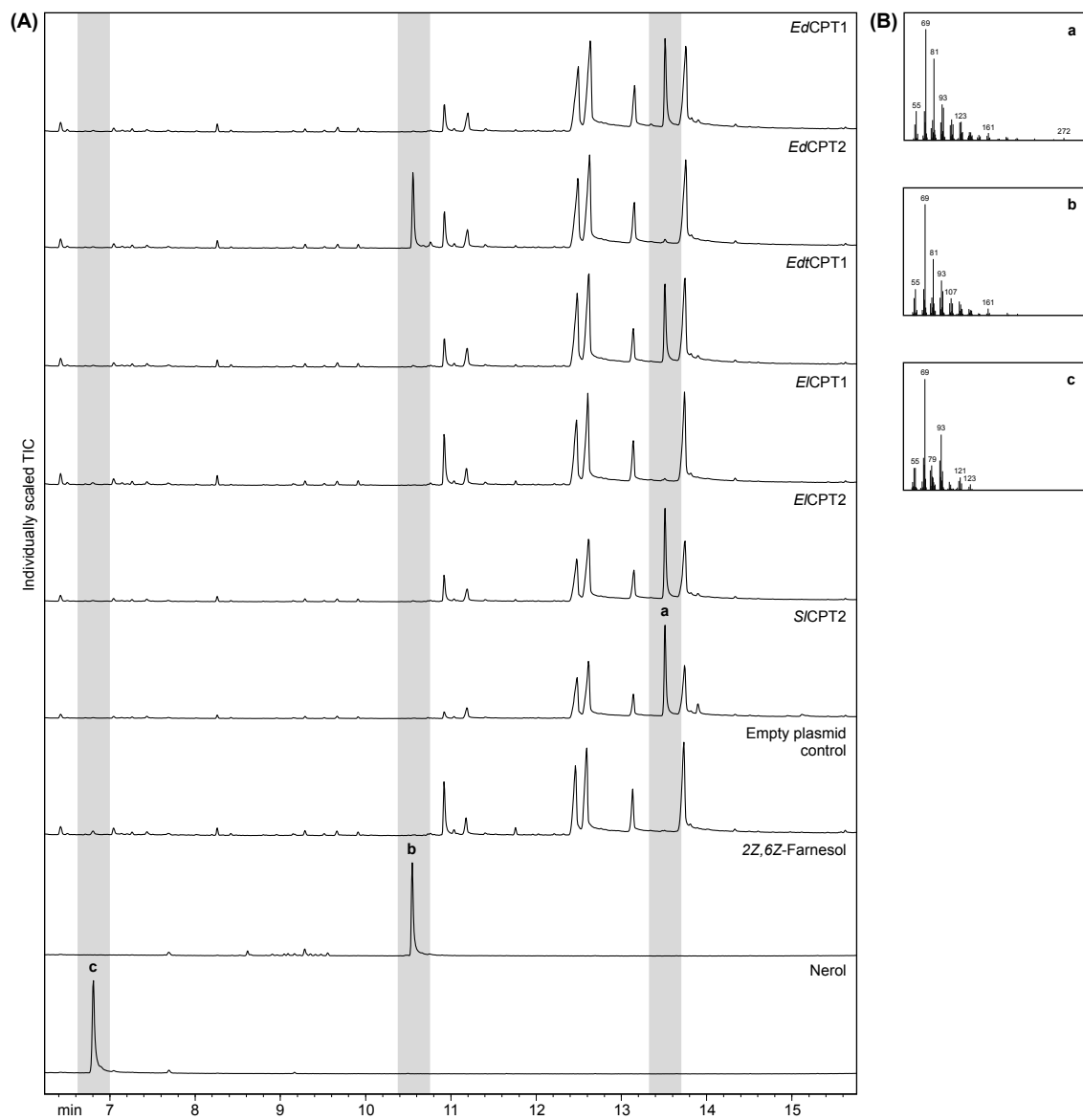

Figure S4

Supplement: Supplementary file 5 — Additional file 5: Figure S4. (A) In vivo functional characterisation of Eremophila CPTs. GC-MS chromatograms of hexane extracts of E. coli cultures treated with alkaline phosphatase. (B) Mass spectra of prenyl alcohols: nerylnerol (a), (2Z,6Z)-farnesol (b) and nerol (c). [file 12870_2020_2293_MOESM5_ESM.pdf]

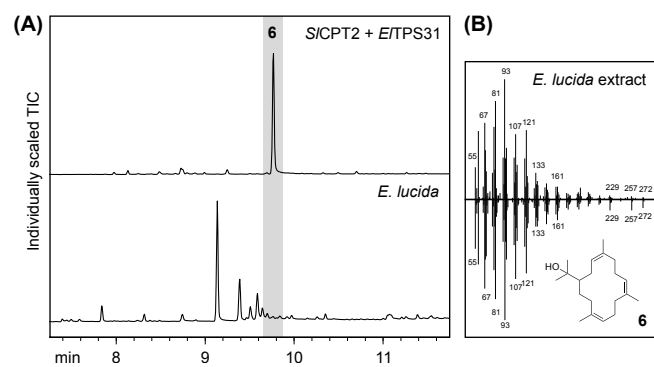

Figure S5

Supplement: Supplementary file 6 — Additional file 6: Figure S5. (A) GC-MS analysis of a hexane extract of E. lucida leaves. Only 6, the product of ElTPS31, was detected in the plant. Comparison trace is of an extract of N. benthamiana leaves transiently expressing ElTPS31 with NNPPS (SlCPT2). (B) Mass spectrum of 6 produced by heterologous expression in N. benthamiana compared to E. lucida leaf extract. [file 12870_2020_2293_MOESM6_ESM.pdf]

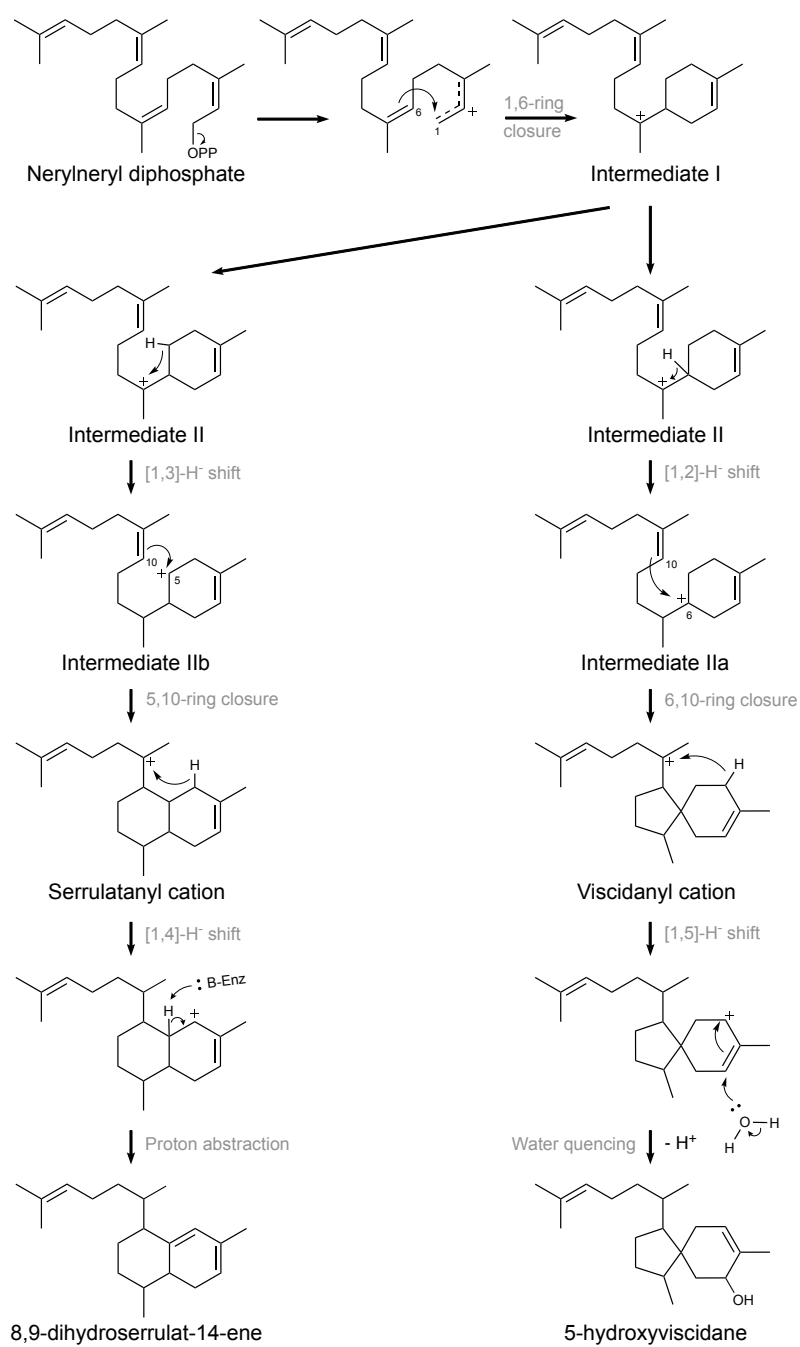

Figure S6

Supplement: Supplementary file 7 — Additional file 7: Figure S6. Proposed reaction pathways catalysed by EdTPS22/EdtTPS4 to 8,9-dihydroserrulat-14-ene and ElTPS3 to 5-hydroxyviscidane from nerylneryl diphosphate. [file 12870_2020_2293_MOESM7_ESM.pdf]
